# *TP53* intron 1 hotspot rearrangements are specific to sporadic osteosarcoma and can cause Li-Fraumeni syndrome

**DOI:** 10.18632/oncotarget.3115

**Published:** 2015-02-25

**Authors:** Sebastian Ribi, Daniel Baumhoer, Kristy Lee, Edison, Audrey S.M. Teo, Babita Madan, Kang Zhang, Wendy K. Kohlmann, Fei Yao, Wah Heng Lee, Qiangze Hoi, Shaojiang Cai, Xing Yi Woo, Patrick Tan, Gernot Jundt, Jan Smida, Michaela Nathrath, Wing-Kin Sung, Joshua D. Schiffman, David M. Virshup, Axel M. Hillmer

**Affiliations:** ^1^ Cancer Therapeutics & Stratified Oncology, Genome Institute of Singapore, Singapore 138672, Singapore; ^2^ Bone Tumor Reference Center at the Institute of Pathology, University Hospital Basel, CH-4003 Basel, Switzerland; ^3^ Clinical Cooperation Group Osteosarcoma, Helmholtz Zentrum Muenchen, German Research Center for Environmental Health, 85764 Neuherberg, Germany; ^4^ Department of Pediatrics and Oncological Sciences, Huntsman Cancer Institute, University of Utah, Salt Lake City, UT 84112, USA; ^5^ Duke-NUS Graduate Medical School Singapore, Singapore 169857, Singapore; ^6^ Institute for Genomic Medicine, UC San Diego, La Jolla, CA 92830, USA; ^7^ Huntsman Cancer Institute, University of Utah Health Care, Utah, UT 84112, USA; ^8^ Computational & Systems Biology, Genome Institute of Singapore, Singapore 138672, Singapore; ^9^ Personal Genomics Solutions, Genome Institute of Singapore, Singapore 138672, Singapore; ^10^ Cancer Science Institute of Singapore, National University of Singapore, Singapore 117599, Singapore; ^11^ Department of Pediatrics and Wilhelm Sander Sarcoma Treatment Unit, Technische Universität München and Pediatric Oncology Center, 81675 Munich, Germany; ^12^ School of Computing, National University of Singapore, Singapore 117417, Singapore

**Keywords:** *TP53*, Li-Fraumeni syndrome, osteosarcoma, cancer genomics, structural variations

## Abstract

Somatic mutations of *TP53* are among the most common in cancer and germline mutations of *TP53* (usually missense) can cause Li-Fraumeni syndrome (LFS). Recently, recurrent genomic rearrangements in intron 1 of *TP53* have been described in osteosarcoma (OS), a highly malignant neoplasm of bone belonging to the spectrum of LFS tumors. Using whole-genome sequencing of OS, we found features of *TP53* intron 1 rearrangements suggesting a unique mechanism correlated with transcription. Screening of 288 OS and 1,090 tumors of other types revealed evidence for *TP53* rearrangements in 46 (16%) OS, while none were detected in other tumor types, indicating this rearrangement to be highly specific to OS. We revisited a four-generation LFS family where no *TP53* mutation had been identified and found a 445 kb inversion spanning from the *TP53* intron 1 towards the centromere. The inversion segregated with tumors in the LFS family. Cancers in this family had loss of heterozygosity, retaining the rearranged allele and resulting in *TP53* expression loss. In conclusion, intron 1 rearrangements cause p53-driven malignancies by both germline and somatic mechanisms and provide an important mechanism of *TP53* inactivation in LFS, which might in part explain the diagnostic gap of formerly classified “*TP53* wild-type” LFS.

## INTRODUCTION

Germline mutations in the *TP53* tumor suppressor gene cause Li-Fraumeni syndrome (LFS), an autosomal dominantly inherited predisposition syndrome to various cancers, including osteosarcoma (OS) [1, 2]. *TP53* coding mutations can be identified in 70% of classic LFS families [3] leaving a significant proportion of LFS cases with an unknown genetic basis. The vast majority of *TP53* mutations in LFS, OS and other tumors are point mutations dominated by missense mutations [4]. Larger germline deletions encompassing the entire *TP53* gene together with neighboring genes have been described to correlate with developmental delay [5]. Partial deletions of *TP53* have been found to be associated with LFS suggesting that the partial loss of *TP53* has a different functional outcome than the entire deletion of the gene [5]. Some genomic structural variations (SVs) have been described that can affect *TP53* function. These SVs are either deletions, which were identified by PCR based methods or comparative genome hybridization, that affect the *TP53* gene in LFS patients [5, 6], or rearrangements in intron 1 of *TP53* which initially have been identified by Southern blot in OS [7–9]. Recently, whole-genome sequencing of tumors from 32 OS patients showed cancer-specific *TP53* rearrangements in > 50% of patients [10].

p53 is a DNA-damage response protein [11] and its inactivation could be expected to result in further genomic instability [12]. Mutations of *TP53* are among the most common defects associated with human cancer in general. Given the large number of *TP53* point mutations which have been identified in the majority of cancer types, it is surprising that *TP53* intron 1 rearrangements have only been found in OS [7–10]. Since exome sequencing does not allow the identification of copy number neutral genome rearrangements with intergenic or intronic breakpoints, it is possible that *TP53* intron 1 rearrangements have been missed in many studies. In addition, the suggested specificity of *TP53* intron 1 rearrangements for OS is based on screens of a limited number of samples [7–9]. Further, it seems possible that *TP53* intron 1 rearrangements do not only contribute to sporadic OS but also to LFS, which is driven by germline *TP53* mutations. In the present study, we analyze the nature of *TP53* intron 1 rearrangements, screen the to date largest collection of OS and other tumor types for such rearrangements, describe the identification of a *TP53* intron 1 disrupting germline inversion in a four generation LFS family and characterize this locus and *TP53* activity in tumors of this family.

## RESULTS

### Characterization of recurrent rearrangement points in intron 1 of *TP53*

**Figure 1:**
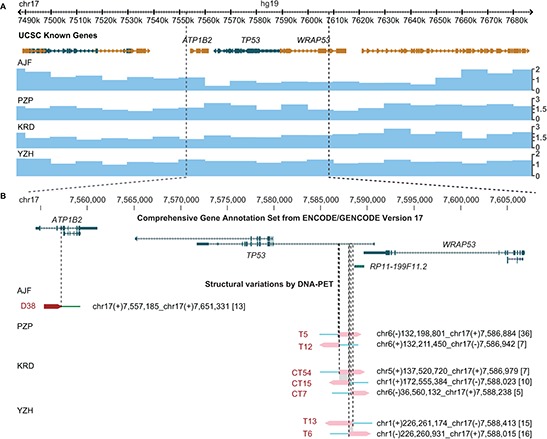
Translocation hotspot in intron 1 of *TP53* in OS samples **(A)** Genes derived from the UCSC known genes database [43] (top) and copy number information derived from DNA-PET sequencing data of four OS samples (blue tracks, bottom) are shown in the Genome Browser. Genes transcribed from the plus strand are represented in gold, genes transcribed from the minus strand are represented in green. Boxes indicate exons, barbed lines indicate introns. The *TP53* locus for patients PZP, KRD, and YZH has a copy number of two while patient AJF shows loss of one copy. **(B)** Enlargement of gene (top) and breakpoint (bottom) view of the *TP53* locus. GENCODE transcripts with unresolved problems have been excluded. Note that *TP53* is transcribed on the minus strand (from right to left). Mapping regions of DNA-PET sequence tags which represent a rearrangement are shown as dark red (5′-tags) and pink (3′-tags) arrow heads with the predicted breakpoint being located at the tip of the dark red and the base of the pink arrow heads (dashed lines). SV identifiers are in red letters, predicted breakpoint locations and connections are indicated for each rearrangement in black letters. Numbers in squared brackets indicate number of PETs which connect the two genomic regions of a SV (dPET cluster size). Shaded in gray are stretches of identical sequences for both breakpoint sides.

We analyzed the genome structures of four pre-therapeutic OS using DNA paired-end tag sequencing (DNA-PET), a genome-wide mate-pair sequencing approach [13–15] and predicted 434, 289, 348 and 420 SVs, respectively, to be somatically acquired (Supplementary Tables S1–S6, Figures S1A and S1B, S2 and S3A, S3B and S3C). We identified seven breakpoints within a small region of intron 1 of *TP53* in three OS tumors (Figure 1, Figure S4 and Supplementary Table S7) and the fourth (AJF) had a 94 kb deletion that included the entire *TP53* gene as well as neighboring genes (Figure 1A and 1B). Tumor YZH showed a balanced translocation between *TP53* intron 1 and chromosome 1. The sequence of the breakpoints showed the presence of the same 555 bp and 293 bp of the *TP53* and chromosome 1 loci, respectively, on both sides of the translocations (Supplementary Figure S5A and S5B). Tumor PZP had a 12.5 kb inverted insertion originating from chromosome 6 containing *ENPP1* exons 19 to 25 including the stop codon (Supplementary Figure S6A and S6B). In addition, the *TP53* intronic sequences on both sides of the insertion overlapped by 59 bp suggesting that a similar mechanism was responsible for the translocations in both YZH and PZP. Tumor KRD had complex inter-chromosomal translocations with the three different partner chromosomes 1, 5 and 6 (Figure 1B) implying that these are three independent events. At least one event had to be non-clonal meaning that two or three independent clones with structural rearrangements in *TP53* intron 1 underlie this tumor. The translocation breakpoints in intron 1 of *TP53* with chromosomes 1 and 6 were only 45 bp apart with an overlap of 46 bp of the intron 1 sequence. The overlap and orientations were compatible with one event of similar mechanism as for tumors YZH and PZP. In contrast, the DNA-PET mapping regions of the chromosome 5 translocation suggest that this rearrangement occurred on the other allele of *TP53* or in an independent clone (Figure 1B).

Centromeric of the breakpoint cluster region (2.5 kb of its center towards exon 1 of *TP53*) data of the Encyclopedia of DNA Elements (ENCODE) [16] show strong signals of open chromatin and active enhancers. It seems possible that the open chromatin state and/or the active transcription of *TP53* contribute to the rearrangement mechanism. Six of the seven breakpoints were located within long interspersed elements (LINE), and the seventh breakpoint was within a short interspersed element (SINE). While the breakpoint partner sites do not show enrichment for LINE or SINE sequences, it is striking that five of the seven partner breakpoints also have strong signals of open chromatin within a region of 10 kb (Supplementary Figures S7A and S7B, and S8 to S12).

In two of the three tumors with *TP53* intron 1 rearrangements, the breakpoint locations predicted gene fusions forming *TP53-ENPP1-TP53* and *SUCO-TP53* (Supplementary Table S7). Interestingly, both fusion gene partners are involved in bone biology. *ENPP1* has been shown to be a key regulator of ossification [17]. *SUCO* (SUN domain containing ossification factor) is an essential regulator of postnatal osteoblast maturation [18]. Furthermore, we found both genes expressed in a collection of in-house established OS cell lines (unpublished data). The expression of the rearranged genes in bone supports the idea that active expression might mechanistically contribute to the translocations.

### Somatic *TP53* rearrangements are a frequent phenomenon specific for OS

**Figure 2:**
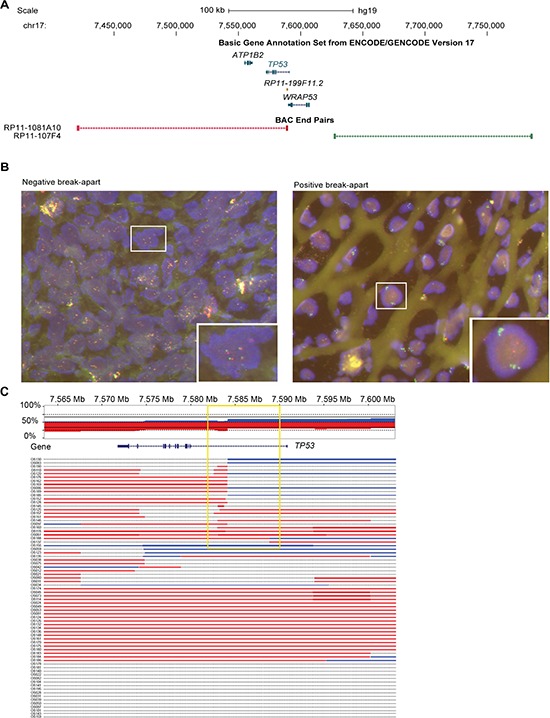
Translocation hotspot in intron 1 of *TP53* in OS samples **(A)** Location of BAC clones which have been selected for FISH relative to *TP53* and immediate neighbouring genes. Other genes of the track have been deleted for clarity. Color code matches fluorophore of FISH analysis shown in B. **(B)** Examples of a negative and positive break-apart signal of two color FISH which has been used to screen 267 formalin fixed and paraffin embedded (FFPE) OS samples and 141 other bone-forming tumors. **(C)** Copy number overview of 73 OS tumors at the *TP53* locus based on CytoScan array analysis. Top panel shows the cumulative copy number across all samples with red indicating loss and blue gain in copy number. Lower panel shows the copy number gains and losses for each of the 73 OS tumors individually. Note the changes in copy number within intron 1 of *TP53* detectable in 23 cases (yellow box).

We designed a break-apart FISH test using probes flanking the *TP53* gene (Figure 2A) and investigated a series of 215 pre-therapeutic OS samples arranged on a tissue microarray (TMA). We found 11% (23 out of 215) of the cases to have rearrangements at the *TP53* locus (FISH break-apart positive; Figure 2B). Of note, in all 23 FISH positive cases, both alleles showed the break-apart signal. However, FISH positive patients did not differ from negative patients in terms of overall-survival (*p* = 0.6), event-free survival (*p* = 0.7), occurrence of metastases, or response to neoadjuvant chemotherapy (Table 1 and Supplementary Tables S8 and S9). Rearrangements at this locus nevertheless appear to be a recurrent finding in OS. To test whether the *TP53* rearrangement also occurred in other bone-forming tumors that sometimes can be difficult to distinguish histologically from OS in small biopsies, we analyzed another series of 124 bone-forming tumors and tumor-like lesions using our FISH assay. None of these cases showed evidence of *TP53* rearrangement. To further exclude *TP53* intron 1 rearrangements in other tumor types we used our FISH assay to analyze an additional 966 tumors on a TMA (Supplementary Tables S10 and S11). None of the 966 tumors showed a break-apart signal suggesting the somatic *TP53* intron 1 rearrangements represent a specific finding in OS.

**Table 1:** Characteristics of OS patients

	OS TMA		OS fresh frozen	
	All	FISH positive (out of 215 evaluable)	All	Intron 1 CN change
**Gender**	254/267 (95.1%)	23/23 (100%)	73/73 (100%)	24/24 (100%)
male	129	9	39	13
female	125	14	34	11
**Age at diagnosis**	258/267 (96.6%)	23/23 (100%)	73/73 (100%)	24/24 (100%)
average	24.3 years	17 years	17.3 years	18.6 years
median	17 years	15 years	15 years	15.5 years
range	4–88 years	6–48 years	3–56 years	5–56 years
**Observation period**	259/267 (97%)	23/23 (100%)	72/73 (98.6%)	24/24 (100%)
average	61.6 months	46.6 months	73.9 months	64.6 months
median	35 months	24 months	66.5 months	64 months
range	0–287 months	0–179 months	0–205 months	2–196 months
**Response to neoadjuvant treatment**	180/267 (67.4%)	19/23 (83%)	66/73 (90.4%)	21/24 (87.5%)
good (< 10% viable tumor)	102	14	35	10
poor (≥ 10% viable tumor)	78	5	31	11
**Metastases**	267/267 (100%)	23/23 (100%)	65/73 (89%)	19/24 (79.2%)
yes	101	9	39	10
no	166	14	26	9
**Survival**	259/267 (97%)	23/23 (100%)	72/73 (98.6%)	24/24 (100%)
alive	174	18	55	16
deceased	85	5	17	8
**TP53 immunhisto-chemistry**	212/267 (79.4%)	19/23 (83%)		
negative	170	15		
positive	42	4		
Location		23/23 (100%)		24/24 (100%)
femur		14		12
tibia		5		8
jaws		2		-
humerus		1		1
fibula		1		1
other		-		2

OS TMA series / OS Fresh Frozen series: number of evaluable cases / total number of cases (percentage) CN, copy number

To further validate our findings by another platform, we analyzed an independent set of 73 pre-therapeutic fresh-frozen OS samples for copy number alterations (CNAs) using CytoScan® high density arrays. We found that 74% (54 out of 73) of the OS samples had alterations affecting the *TP53* gene. Amongst these alterations, 23 showed transition points into copy number losses (*n* = 17) or gains (*n* = 3) or abrupt transitions from losses into gains (*n* = 3) in intron 1 of the gene (32% of total samples, 23 out of 73, Figure 2C). Again, the rearrangements did not correlate with any clinico-pathological parameters.

### *TP53* intron 1 rearrangement in a family with LFS

**Figure 3:**
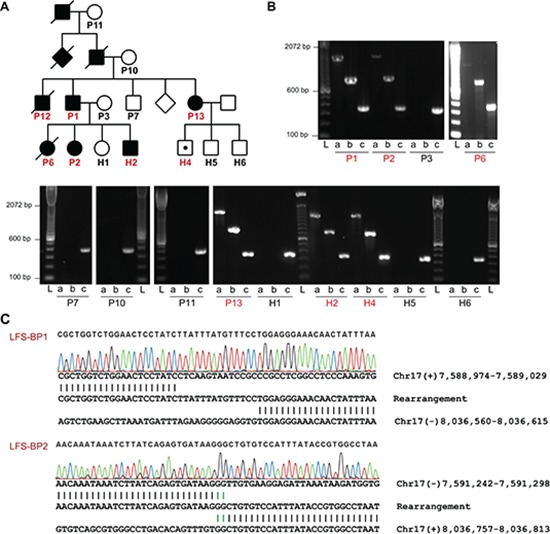
*TP53* intron 1 rearrangement in a family with LFS **(A)** Pedigree of a family with LFS. Squares and circles represent males and females, respectively. Filled symbols indicate individuals with early onset cancer. Symbols with a diagonal line indicate that the individual was deceased at the time of recruitment. Diamonds represent several additional family members whose gender should not be disclosed. Individuals of whom DNA samples have been obtained are numbered with individuals tested positive for *TP53* exon 1 deletion by MLPA in red. *TP53* rearrangement carrier without cancer at the age of 10 years is indicated by a white symbol with a black dot. **(B)** PCR analysis of positive control and *TP53* intron 1 rearrangement points (LFS-BP1 and LFS-BP2) of family members of whom high quality DNA was available. DNA quality of P12 did not allow PCR amplification, including positive control (not shown). a, LFS breakpoint 1 (BP1); b, LFS breakpoint 2 (BP2); c, positive control amplicon at the RNA polymerase II locus (POLR2A); sample IDs correspond to A with affected individuals in red. L, ladder. **(C)** Sanger sequencing of rearrangement points LFS-BP1 and LFS-BP2 at the *TP53* locus of P2. Micro homologies between the two participating break point regions are illustrated by green vertical lines. Genomic coordinates are based on NCBI build 37.

**Figure 4:**
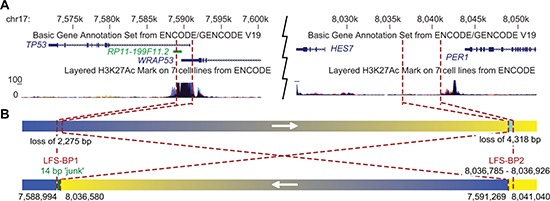
Inversion of 445 kb affecting intron 1 of *TP53* in a family with LFS **(A)** University of California, Santa Cruz (UCSC) genome browser view [43] of the *TP53* locus with representative *TP53* and *WRAP53* transcripts, and H3K27Ac binding information of the ENCODE project [44]. Breakpoint locations are indicated by red dashed lines. **(B)** Schematic view of a 445 kb inversion affecting *TP53* in LFS family. The telomeric rearrangement points of the inversion are located in the breakpoint cluster region in intron 1 of *TP53*. Arrows indicate genomic orientations pointing towards higher coordinates. Blue, *TP53* locus; yellow, *HES7*/*PER1* locus; chromosome 17 coordinates of breakpoints are indicated below.

Since mutations of *TP53* are associated with LFS it seemed possible that *TP53* intron 1 rearrangements could constitute a previously underappreciated category of alterations that can cause LFS. We revisited an LFS family with 12 affected members with cancer across four generations in which we previously had been unable to identify a coding *TP53* mutation or a co-segregating, potentially damaging and disease-causing alteration based on exome sequencing of patients P1, P2 and P13 (Figure 3A and data not shown). Copy number analysis of DNA from blood of patients P1 and P13 by single nucleotide polymorphism (SNP) array revealed evidence for an approximately 2.5 kb deletion in intron 1 of *TP53* including exon 1 (Supplementary Figure S13 and Table S12). However, we were not able to amplify the fusion point of a deletion by PCR. We then used a custom sequence capture assay for targeted paired-end sequencing of the *TP53* locus to search for a rearrangement point. We identified a 445 kb inversion spanning from the breakpoint cluster region in intron 1 of *TP53* towards the centromere (upstream of *TP53*) with a loss of 2,275 bp of intron 1/exon 1 of *TP53* (Figure 4A and 4B). The lost sequence was in agreement with the deletion identified by SNP arrays. In the adult family members tested, we found this specific rearrangement co-segregating with the disease, implicating the rearrangement as the causative alteration (Figure 3B and 3C).

Twelve different protein isoforms of *TP53* (p53, p53β, −γ, Δ40p53α, −β, −γ, Δ133p53α, −β, −γ, Δ160p53α, −β, −γ) can be generated by alternative splicing, alternative promoter usage, and alternative initiation sites of translation which have different functional properties (reviewed in [19, 20]). A weak promoter is located just upstream of exon 1, a strong promoter 1 kb downstream of exon 1 and a third promoter at exon 5 [19, 21]. Transcripts for p53 (full length) and the Δ40p53 isoforms contain the non-coding exon 1. The intron 1 rearrangements disconnect the exon 1 of *TP53* and the two first promoters from the remaining gene body. To investigate the impact of *TP53* rearrangements on *TP53* expression, we obtained RNA from blood of LFS patients H2 and P13, OS lung metastasis of H2 and a cell line derived from the lung adenocarcinoma of P13.

**Figure 5:**
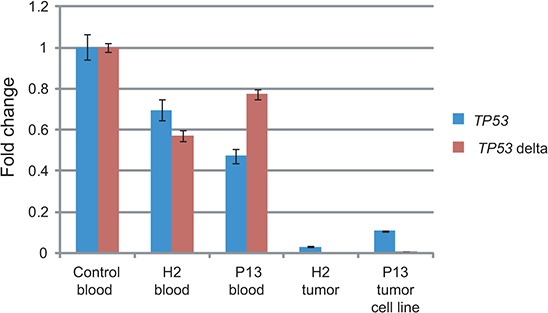
Tumors of LFS family show impaired *TP53* transcription RNA was extracted from blood from one unrelated control individual, *TP53* intron 1 rearrangement carriers of the LFS family H2 and P13, OS lung metastasis of H2 and a lung adenocarcinoma derived cell line of P13. qRT-PCRs targeting all twelve *TP53* isoforms (*TP53*) and short transcripts encoding for the N-terminus lacking isoforms Δ133p53 and Δ160p53 (*TP53* delta) were performed in triplicates. Quantification cycle values (Cq) were normalized to *GAPDH* expression and are shown as fold change relative to control (y-axis). Error bars represent standard deviations. One representative of two experiments.

We performed quantitative reverse transcription polymerase chain reactions (qRT-PCRs) targeting transcripts encoding for the twelve described p53 isoforms and for isoforms Δ133α, −β, −γ and Δ160α, −β, −γ (*TP53* delta), respectively, and found a reduction of transcripts by 23–53% for the blood of H2 and P13 where the rearrangement was in a heterozygous state, and a reduction by 89–100% for the OS lung metastasis of H2 and a cell line derived from the lung adenocarcinoma of P13 (Figure 5). This implies that the rearrangements result in a loss of *TP53* transcription and function rather than a switch to reported transcripts lacking exon 1.

**Figure 6:**
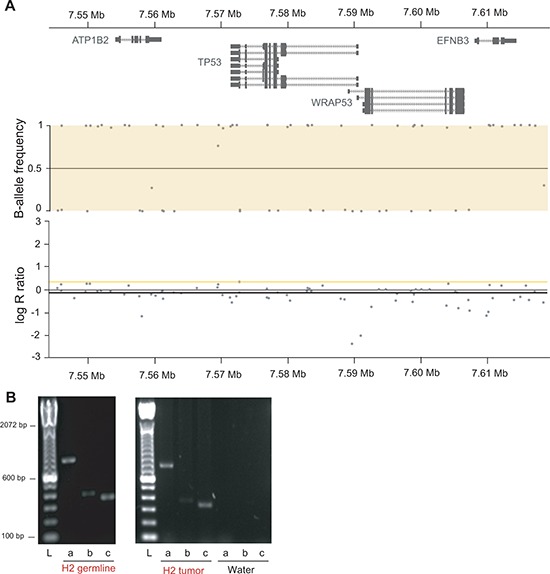
Tumors of LFS family show LOH at the *TP53* locus **(A)** OncoScan array derived copy number and allele frequency plots of the *TP53* locus of OS lung metastasis of H2 show copy number neutral LOH. Chromosome 17 at 7.544 Mb to 7.618 Mb is shown with gene track (top), copy number (middle) and allele frequency (bottom). Y-axis of copy number is copy number ratio compared to reference (normal); y-axis of allele frequency shows homozygous allele calls for all single nucleotide polymorphisms in the window with values close to 0 and 1, respectively. **(B)** Semi quantitative PCR of LFS-BP2 (a), the wild type allele at the BP2 locus (b) and RNA polymerase II (positive control, c) of H2 germline DNA (30 PCR cycles) and H2 OS lung metastasis (28 PCR cycles) is shown. L, ladder.

To test for deletions of the second allele in tumors, copy number analysis using OncoScan FFPE Express (Affymetrix, Inc.) were performed on tumor samples of H2 (two OS lung metastases that developed six months apart), P1 (undifferentiated pleomorphic sarcoma) and P13 (lung adenocarcinoma and meningioma). Loss of heterozygosity (LOH) at the *TP53* locus occurred in all five investigated tumors (Figure 6A and Supplementary Figures S14 and S15). Semi quantitative genomic PCR for LFS-BP2 showed a stronger signal for the LFS breakpoint than for the non-rearranged allele suggesting a duplication of the rearranged allele and loss of the non-rearranged allele with the weak PCR signal derived from stroma contamination (Figure 6B). Our findings suggest LOH as a frequent mechanism for *TP53* inactivation after an initial intron 1 rearrangement.

## DISCUSSION

### Rearrangement hotspot in *TP53* intron 1

More than 20 years ago, rearrangements in *TP53* in OS were identified by Southern blot [7–9]. Although based on a small number of samples, these rearrangements were thought to be specific for OS. Recently, *TP53* rearrangements have been rediscovered in OS in more than 50% of 32 OS by whole-genome sequencing at a higher resolution [10]. In our study, we also found *TP53* intron 1 rearrangements to be the most recurrent focal rearrangement point in four OS samples. We found evidence for *TP53* rearrangements in 11% (23 out of 215) of OS samples by FISH and copy number changes in intron 1 of *TP53* in 32% (23 out of 73) of OS samples by CytoScan arrays. Importantly, there were no rearrangements found in other bone-forming tumors (0 out of 124) by FISH analysis. Due to the low resolution of the bacterial artificial chromosome (BAC) probes of the FISH experiments which were 169 kb and 159 kb in size, respectively, the 23 detected rearrangements might include translocations outside intron 1 of *TP53*. However, we did not observe other rearrangements which could result in the ‘break-apart’ FISH signal in the whole-genome DNA-PET data of the four sequenced OS samples. Similarly, Chen *et al.* also found the majority of breaks in intron 1 [10], suggesting that most of the rearrangements detected by FISH were due to intron 1 rearrangements. In addition, the FISH analysis did not have the resolution to identify small insertions as discovered in tumor PZP or deletions of similar sizes, thus the actual frequency of *TP53* intron 1 rearrangements might be underestimated. Further, we found *TP53* intron 1 rearrangements in OS by CytoScan analysis which has the resolution to locate the breakpoints to the first intron of *TP53*. The different frequencies of identified *TP53* rearrangements by FISH and CytoScan arrays might be explained by the lack of sensitivity of the FISH assay for the identification of small rearrangements. The differences of *TP53* intron 1 rearrangement frequencies between our study and the report of Chen and colleagues may be due to different analysis protocols or sampling biases.

### *TP53* intron 1 rearrangement mechanism

Three main types of mechanisms for genome rearrangements have been established: homologous recombination, replication-based mechanisms, and non-replicative non-homologous repair [22, 23]. The *TP53* intron 1 locus does not show significant sequence similarity with the seven translocation partner sites, arguing against a homology driven rearrangement mechanism. A replication coupled mechanism seems to be unlikely as well since a) it would require two replication fork invasions occurring in parallel at the same locus, b) it would involve two non-homologous chromosomes and replication-based mechanisms such as fork stalling and template switching (FoSTeS), which to the best of our knowledge have been described only for intra-chromosomal rearrangements, and c) we did not find evidence for rearrangement points which ‘switch back’ to the original *TP53* chromosome 17. The rearrangement points of non-replicative non-homologous repair, with the subgroups non-homologous end joining (NHEJ) and alternative end joining, are characterized by the absence of large homology but the presence of micro-homology of a few base pairs, blunt end joining or the insertion of small stretches of ‘junk’ DNA of unknown origin.

The *TP53* intron 1 region does not seem to be compatible with these common mechanisms of genome rearrangements or might be classified as a subcategory of NHEJ with sequence duplication. What we found in the three OS tumors with sequenced *TP53* intron 1 rearrangement points is different since both sides of the balanced intron 1 rearrangements contain a stretch of identical sequence of 46 bp to 555 bp. We therefore suggest a mechanism where a double strand break with long single stranded DNA overhangs occurs (Supplementary Figure S16A to S16G). The single stranded DNA overhangs might get filled in by the DNA repair machinery allowing blunt end fusions with other double strand breaks. Remarkably, all three OS tumors in which we initially found the *TP53* rearrangements share this unique feature of breakpoint sequences suggesting that they were caused by the same mechanism. The break point regions of the *TP53* locus and the translocation partner sites show a pattern of general active chromatin marks and gene expression in OS and/or bone tissue. The open state of the chromatin and/or the active expression of the respective genes may lead or contribute to the formation of the *TP53* intron 1 specific rearrangements.

### Somatic SVs in *TP53* intron 1 are specific to OS

We assayed a comprehensive tumor collection of more than ten tissue types with a total of 1,090 non-OS tumors and 215 OS and show that the mechanistic event of the somatic rearrangements in *TP53* is highly specific to OS. In contrast, our findings in the LFS family indicate that *TP53* intron 1 can also occur in the germline and once such a SV is present as a germline alteration it can give rise to not only OS but multiple types of cancer, including adenocarcinoma, meningioma, astrocytoma, colon cancer, basal cell and squamous cell carcinoma of the skin. Interestingly, none of these tumor types were positive in our FISH TMA assay (*n* = 54, *n* = 14, *n* = 11, *n* = 62, *n* = 23 and *n* = 10, respectively) indicating that the mechanistic occurrence of *TP53* intron 1 rearrangements in the soma is specific for the osteoblast lineage, the believed cell of origin for OS [24]; however, the pro-cancer effect of such rearrangements supports tumor growth in many other tissues. Similarly, *TP53^−/−^* and *TP53^+/−^* knockout mice develop not only OS but also lymphoma, carcinoma and testicular cancer [25]. Again, our FISH analysis of tumors of these types did not show evidence for somatic *TP53* intron 1 rearrangements (*n* = 33, *n* = 566, *n* = 33, respectively). It therefore seems plausible that the OS specificity of the *TP53* intron 1 SVs is based on the mechanism of the rearrangement that leads it to occur only in osteoblast lineage with observable frequency, rather than OS being the specific consequence of the alteration. Cell lineage specific DNA replication properties or transcriptional processes might be responsible for the remarkable specificity of the described rearrangements.

### Other genes at the rearrangement hotspot

It remains possible that the intron 1 rearrangements affect other genes at this locus which could result in pro-malignant effects. *RP11–199F11.2* (Hp53int1 or D17S2179E) is a non-spliced, probably non-coding transcript in intron 1 of *TP53* which is transcribed in the same orientation as *TP53* (Figure 1B). It has been identified by a targeted cDNA library screen but its function is unknown [26]. Further, exon 1 of *TP53* overlaps with *WRAP53*, a gene which is oriented in antisense relative to *TP53*, on the plus strand of chromosome 17, that has been found to upregulate *TP53* transcripts. Downregulation of *WRAP53* is reported to lead to significant suppression of p53 induction in response to DNA damage [27]. *WRAP53* protein has been characterized as an essential protein for the localization and processing of nuclear ribonucleoproteins [28, 29].

### Germline SVs in *TP53* intron 1 can cause LFS

We found germline rearrangements in the *TP53* intron 1 hotspot likely to be causative for LFS in a large family. Our findings are in agreement with the second hit model for tumor suppressor genes where all 23 FISH positive OS showed two break-apart signals and the LFS family had the rearrangement in a heterozygous state in the germline followed by LOH in all tumors. Our qRT-PCR experiments suggest that the overall transcription of *TP53* is lost upon intron 1 rearrangement but it remains possible that in certain tissue/tumor contexts or depending on regulatory elements in the rearrangement partner sites, such rearrangements could cause a shift towards the expression of the shorter isoforms Δ133p53 and Δ160p53. In contrast to p53, Δ133p53 is defective in promoting apoptosis [21]. The comparison of *TP53* transcripts in breast tumor versus normal tissues revealed obvious differences with Δ133p53 being expressed in 24 out of 30 breast tumors but not in 8 normal breast tissue samples [21]. This is supported by a clinical study which found Δ133p53 isoforms to be abnormally expressed in renal cancer, suggesting that they play a role in carcinogenesis [30]. Further, Δ133p53α inhibits p53-mediated replicative senescence, promotes cellular proliferation of normal human fibroblasts by inhibiting p21 expression, and represses the expression of miR-34a to regulate p53-mediated senescence [31]. Cotransfection experiments indicated that Δ133p53 has a dominant negative effect on the proapoptotic properties of p53 [21]. p53 forms tetramers to execute its function as a transcription factor. Coimmunoprecipitation experiments showed that Δ133p53 forms a complex with p53 and it is likely that this interaction mediates a negative effect on p53 function of the intact allele by interfering with the tetramer structure [19].

*TP53* coding mutations are identified in 70% of classic LFS families [3]. Germline *TP53* intron 1 rearrangements might be present in some of the remaining 30% of families, suggesting that LFS and Li-Fraumeni-like (LFL) families should be tested for such rearrangements. This could be tested using FISH and *TP53* capture-based sequencing approaches for break point identification as in our study. Only a few cases have been reported where genomic structural alterations, usually deletions, cause cancer syndromes [32–39] and one study where a Robertsonian translocation results in a highly increased risk for childhood acute lymphoblastic leukemia with focal chromosome 21 amplification [40]. We present here a very rare phenomenon of a rearrangement hotspot which can give rise to both somatic rearrangements as well as a germline cancer syndrome. It seems possible that the open chromatin state together with components of the transcription machinery and characteristic LINE sequences underlie the fragility of this locus. The disruption of the gene structure results in loss of p53 function and thereby promotes cancer. In conclusion, intron 1 of *TP53* represents a rare case of a tumor type dependent somatic rearrangement hotspot that can also acquire germline SVs causing a Mendelian inherited cancer syndrome.

## METHODS

### OS patient samples for DNA-PET sequencing

DNA of four treatment naive OS tumors and paired normal blood were obtained from the Biopathology Center (BPC) of the Children's Oncology Group (COG), a cooperative group that includes medical centers in the United States, Canada, Mexico, Australia, New Zealand and selected countries in Europe. Informed consent of the participating patients or legal representatives has been obtained and approval of the respective institutional ethics review boards has been granted.

### DNA-PET libraries construction, sequencing, mapping and data analysis

DNA-PET library construction from 1 to 4 kb fragments of genomic DNA, sequencing, mapping and data analysis were performed as described in [13] with refined bioinformatics filtering as described in [41]. High throughput sequencing by 2 × 35 bp or 2 × 50 bp was performed on SOLiD sequencers (v3plus and v4, respectively) according to the manufacturer's recommendation (Life Technologies). The short reads were aligned to the NCBI human reference genome build 37 (hg19) using Bioscope (Life Technologies).

### Custom sequence capture and breakpoint identification in a LFS family by paired-end sequencing

The *TP53*-containing region chr17:7,520,000–7,680,000 (NCBI build 37) was defined for a custom sequence capture (SeqCap EZ Choice, Roche NimbleGen Inc.). Repetitive regions are usually excluded for sequence capture assays. Since most of the observed breakpoints in OS were in LINE sequences, we forced to include repetitive sequences of the intron 1 region of *TP53* (chr17:7,579,941–7,590,694). Illumina sequencing library was constructed and capturing was performed according to the manufacturer's recommendations and the library was sequenced on an Illumina HiSeq 2000 by 2x50 bases. Reads were mapped to the human reference genome (NCBI build 37) by BWA and read-pairs were filtered of which one read mapped to the *TP53* target region and the paired read mapped outside of that region. Reads of similar mapping pattern were clustered together as described earlier [13] and prioritized for validation by PCR and Sanger sequencing based on cluster size (number of paired-reads with similar mapping patterns). Identified *TP53* breakpoints were screened by PCR in all LFS family members of whom DNA was available. Validation PCRs were conducted using Jumpstart REDAccuTaq LA DNA Polymerase (Sigma-Aldrich) following manufacturer's instructions with 120 ng DNA template. Where sample was insufficient, the genomic DNA was amplified using REPLI-g Mini Kit (Qiagen) and 3 ul of 1:20 diluted amplification product were used as template for validation PCRs. PCRs were performed using the following primer pairs: CTCAAAAGGCC ATCAAAAGG and GTATGGTGGCCTGTTCCTGT (LFS-BP1), GGCTGCTGGGAGTTGTAGTC and AGCT ATCTTGACCCCACACG (LFS-BP2), CCCGAATA GCTGGGATTACA and GCAAGTGCAC AGGAAGATGA (LFS-BP1-wt), GGAGGAATCC TGCATTGTGT and CAGGCTTCAGACCTGTCTCC (LFS-BP2-wt), GCTGCTGGACGTGAGTATGA and AGTTCCAACAATGGCTACCG (positive control primers for POLR2A), and the following cycling conditions: 3 min at 94°C, (20 s at 94°C, 30 s at 58°C, 2 min at 68°C)x15, (20 s at 94°C, 30 s at 55°C, 2 min at 68°C)x20, 5 min at 68°C.

### Tissue samples and patient's characteristics for fluorescence *in situ* hybridization (FISH) and CytoScan screen

All tissue samples were obtained from the archives of the Bone Tumor Reference Center at the University Hospital Basel and the Clinical Cooperation Group Osteosarcoma at the Helmholtz Zentrum Muenchen. Only specimens from patients without prior treatment were included in the study. FISH analyses were performed on nine TMAs comprising OS samples of 267 patients, samples from other bone-forming tumors or tumor-like lesions of 144 patients, and a collection of 1,163 tumors of various types and normal tissue samples. For the CytoScan HD Arrays (Affymetrix, CA, USA) genomic DNA from fresh frozen samples of 73 independent OS patients was used. Full patient's characteristics are presented in Table 1 and Supplementary Tables S8, S10 and S11.

### Dual-color break-apart FISH

BAC clones RP11–1081A10 and RP11–107F4 (BACPAC, Children's Hospital Oakland Research Institute, USA) were nick-translated (Abbott Laboratories, USA) and labeled with fluorescent dUTPs (Enzo Life Sciences, USA) resulting in red labeling of RP11–1081A10 and green labeling of RP11–107F4. The *in situ* hybridisation was performed according to routine protocols. Tumors were considered to have a rearrangement in the 5′ region of *TP53* when at least 10% of cells showed clearly separated green and red hybridisation signals (= FISH positive/break-apart).

### CytoScan HD arrays

Genome-wide CytoScan HD Arrays (Affymetrix, CA, USA) were performed according to the manufacturer's instructions using 250 ng of genomic DNA from each tumor sample. To evaluate copy number variations (CNV), data was processed using the Nexus Copy Number software (Version 7.0, BioDiscovery, CA, USA) with integrated algorithms for segmentation and normalization.

### Clinical characteristics of a four-generation LFS family

All members of the LFS family who contributed samples to this study or legal representatives gave informed consent and are indicated with numbers in Figure 3A. The study was approved by the respective ethical review boards. P1 was diagnosed with colon cancer at age of 22 years, an oligodendroglioma at 34 years, and an undifferentiated pleomorphic sarcoma at 40 years, and basal cell carcinoma and squamous cell carcinoma of the skin, P2 a phyllodes tumor of the breast at 12 years, P6 a rhabdomyosarcoma at 33 months, P12 an OS at 19 years, H2 an OS at 13 years, P13 bilateral ductal carcinoma *in situ* at 32 and 33 years, meningioma at 38 years, and adenocarcinoma of the lung at 40 years. Family members P1, P7, P13, P12, H1, H2, H4, H5 and H6 were analyzed by multiplex ligation-dependent probe amplification (MLPA) (MRC-Holland) according to the manufacturer's recommendation to test for deletions in *TP53* with 2 probe sets in exon 1, and one probe set each in exons 2 – 9, 11, and 12. Both probe sets for exon 1 with probe pair TGTAGCTGCTGGGCTCCGGGGACACT and TTGCGTTCGGGCTGGGAGCGTGCTTTCCACGA (exon 1) and CCATTTCCTTTGCTTCCTCCGGCA and GGCGGATTACTTGCCCTTACTTGTCATGGCGACTGT CCAG (5′ of exon 1) indicated a heterozygous deletion of exon 1 for P1, P13, P12, H2 and H4 but not for P7, H1, H5 and H6. The presence of the deletion co-segregated with affection status except for H4 who was tested positive but had no cancer at the age of 10 years (of note, H4 is being followed with repeat imaging due to suspicious lesion in the brain that could represent the development of an early tumor).

### qRT-PCR of *TP53* in samples of LFS family

RNA was isolated from PAXgene Blood RNA Tubes using the PAXgene Blood miRNA kit (Qiagen). RNA from fresh frozen tumor, H2, and RNA from a tumor derived cell line, P13, were isolated using RNAeasy Mini kit (Qiagen). The one step qRT-PCR was carried out using QuantiTect Probe RT-PCR kit (Qiagen), with a total of 50 ng RNA per PCR. The TaqMan primer/probe set (LifeTechnologies) was used for *TP53* full length (HS01034249) and the PrimeTime primer/probe set (Integrated DNA Technologies Inc.) for transcripts encoding for Δ133p53 and Δ160p53 were designed using sequences from Marcel and colleagues [42]. qRT-PCRs were performed on a Bio-Rad CFX device. Cq values were normalized against *GAPDH* as ΔCq and displayed relative to normal blood control as ΔΔCq as fold-change (2^−ΔΔCq^).

### OncoScan FFPE Express (Molecular Inversion Probe) array

Samples were processed using the Affymetrix OncoScan FFPE Express kit according to the manufacturer's instructions using 80 ng of genomic DNA from each tumor sample. To evaluate copy number variations (CNV) data was processed using the Nexus Copy Number software (Version 7.5, BioDiscovery).

### Sequencing data

Sequences have been submitted to the Short Read Archive (http://trace.ncbi.nlm.nih.gov/Traces/sra/) at the National Center for Biotechnology Information (NCBI) with the study reference number PRJNA244486. LFS array data can be accessed at Gene Expression Omnibus (http://www.ncbi.nlm.nih.gov/geo/) with the study ID GSE64293.

## SUPPLEMENTARY FIGURE AND TABLE




