# Supplementary material for: TP53 intron 1 hotspot rearrangements are specific to sporadic osteosarcoma and can cause Li-Fraumeni syndrome
Source: Oncotarget. 2015 Feb 25;6(10):7727–40. doi: 10.18632/oncotarget.3115 (PMC4480712; doi:10.18632/oncotarget.3115)
Supplement: Supplementary file 1 [file oncotarget-06-7727-s001.pdf]

# SUPPLEMENTARY FIGURES AND TABLES

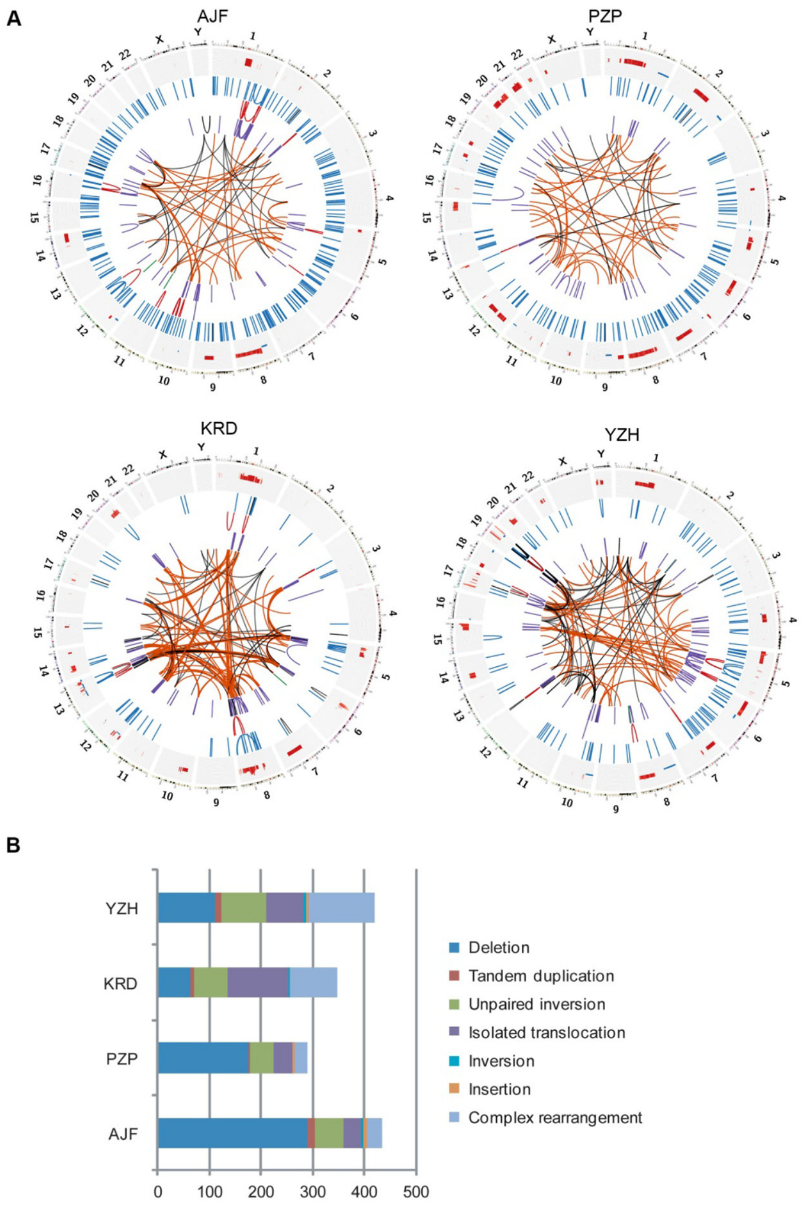


**Supplementary Figure S1: Genome rearrangements of four primary OS analyzed by DNA-PET sequencing. (A)** Genomic SVs enriched for somatic events identified by genome-wide mate-pair sequencing of long DNA fragments of four OS tumors are displayed by Circos [1]. The human genome is arranged in a circle with the chromosome number and the cytogenetic bands displayed in the outer ring, the somatic copy number alterations in the next inner concentric ring (red, amplifications; blue, deletions), followed by deletions, tandem duplications, inversions/unpaired inversions, and in the center of the circle inter-chromosomal rearrangements. Isolated and complex inter-chromosomal rearrangements are represented by orange and black lines, respectively. **(B)** Distribution of SV categories which are enriched for somatic events in the four OS tumors shown in A, X-axis indicates absolute numbers of color coded SV types as described in the legend.

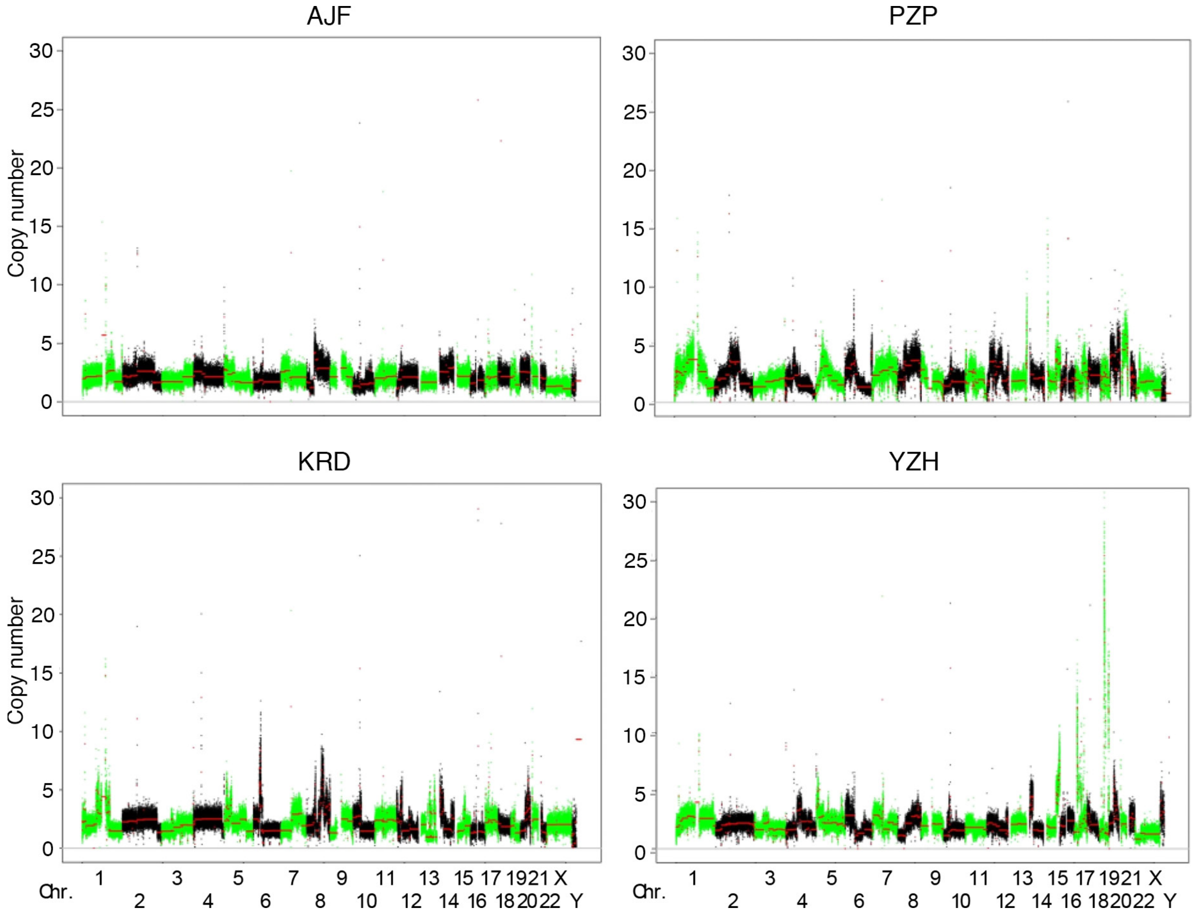


**Supplementary Figure S2: Copy number profile of four primary OS derived from DNA-PET sequencing.** Chromosomes are aligned on the x-axis and copy number values are on the y-axis. All four OS display a large number of copy number alterations.

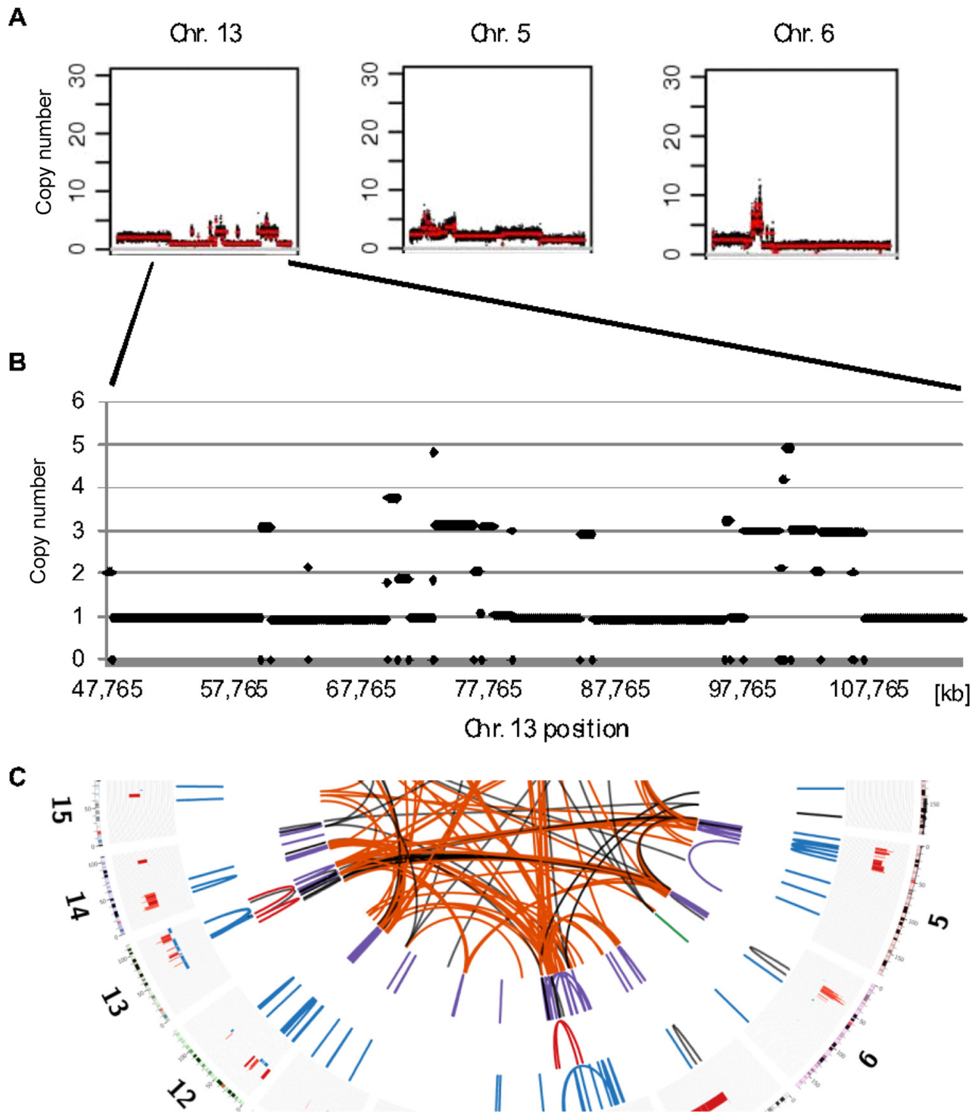


**Supplementary Figure S3: Evidence for chromothripsis in OS tumor KRD. (A)** Copy number profiles of chromosomes 13, 5 and 6 derived from concordant mapping paired-end tag sequencing data relative to simulation control. Chromosomes are aligned on the x-axes and copy number values are on the y-axes. Black dots indicate copy number based on 10 kb windows. Red lines are smoothened copy number across neighboring windows. **(B)** Enlarged copy number profile of chromosome 13 positions 47.7 Mb to 115.1 Mb. Data points are based on 10 kb windows. **(C)** Capture of genomic Circos plot [1] including chromosomes 13, 5 and 6. Information displayed in the concentric rings is as follows from outside to inside: chromosome number; cytogenetic banding, copy number with gains in red and losses in blue; deletions; tandem duplications; inversions/unpaired inversions; inter-chromosomal rearrangements. Color coding for inter-chromosomal SVs is indicated in orange and inter-chromosomal SVs in complex regions in black.

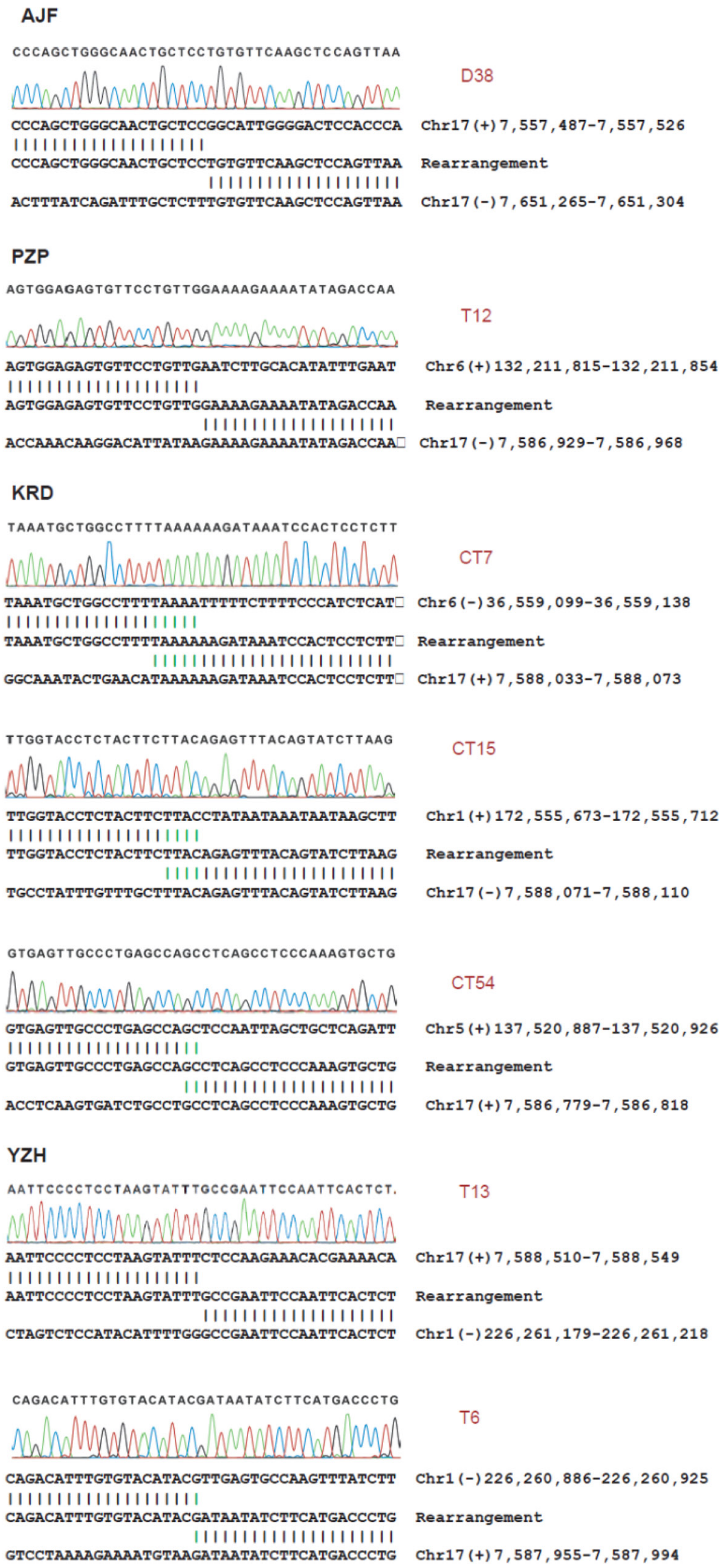


**Supplementary Figure S4: Validation of *TP53* rearrangements by PCR and Sanger sequencing.** Patient IDs are indicated in bold capital letters. Bases matching between the rearrangement and the reference sequences of the participating regions are indicated by vertical lines. Micro homologies between the two participating break point regions are illustrated by green vertical lines. Break point IDs are shown in red.

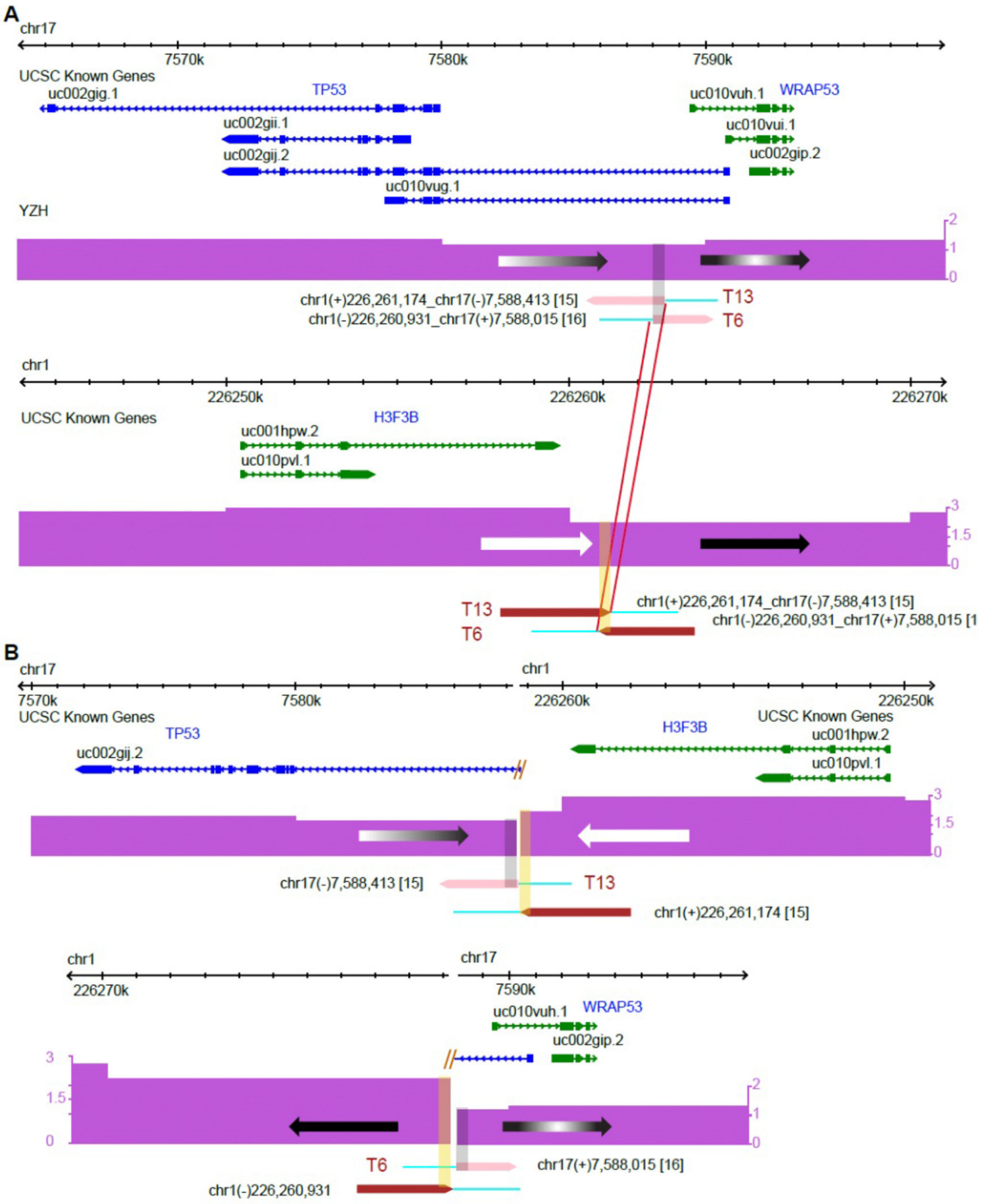


**Supplementary Figure S5: Balanced translocation between intron 1 of *TP53* and a locus on chromosome 1 in OS patient YZH.** **(A)** Chromosome 17 and 1 loci of the human reference genome (hg19) which show the PET mapping regions indicating the balanced translocation between the two loci. Genes showing the major splice variants derived from the UCSC known genes database [2] are shown on top and smoothened copy number information derived from DNA-PET sequencing data is shown at the bottom (purple tracks) in the Genome Browser. Genes transcribed from the plus strand are represented in green, genes transcribed from the minus strand are represented in blue. Boxes indicate exons, barbed lines indicate introns. Mapping regions of 5' and 3' PET clusters and read orientations are indicated by dark red and pink arrow heads, respectively, with turquois lines indicating the side of breakpoint. Gray and yellow shading indicate sequences of 555 bp and 293 bp which are shared by both sides of the balanced translocation product for chromosome 17 and 1, respectively. Red lines indicate connectivity between the two chromosomes. Note that DNA-PET coordinates differ from the exact break point coordinates which have been determined by PCR and Sanger sequencing and which are shown in Table S7. **(B)** Translocation outcome as indicated by DNA-PET truncating *TP53* and duplicating gray and yellow shaded sequences.

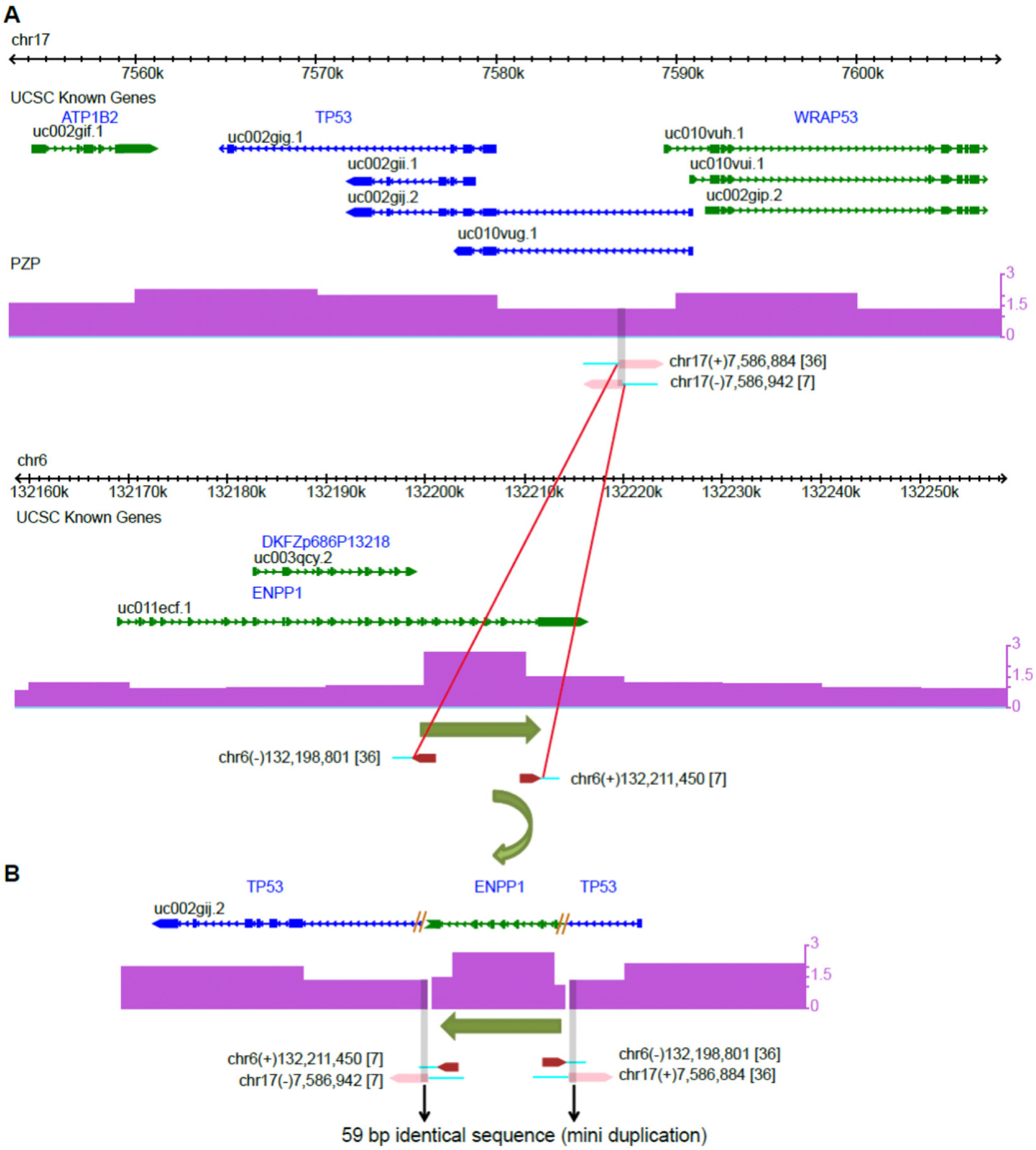


**Supplementary Figure S6: Inverted insertion of 12.5 kb of chromosome 6 into intron 1 of *TP53* in OS patient PZP.** Identification of an inverted insertion originating from chromosome 6 containing *ENPP1* exon 19 to the 5' part of exon 25 into intron 1 of *TP53* by DNA-PET. For more detailed track information, see legend of Supplementary Figure S5, for exact break point coordinates, see Table S7. **(A)** Chromosome 17 and 6 loci of the human reference genome (hg19) which show the PET mapping regions indicating the inverted insertion. **(B)** Insertion outcome as indicated by DNA-PET creating a potential fusion transcript between exon 1 of *TP53* (right blue box) and exon 19 to the first part of exon 25 of *ENPP1*. Since exon 1 of *TP53* is non-coding, and the inserted part of *ENPP1* exon 25 contains a stop codon, the formation of a fusion protein is unlikely.

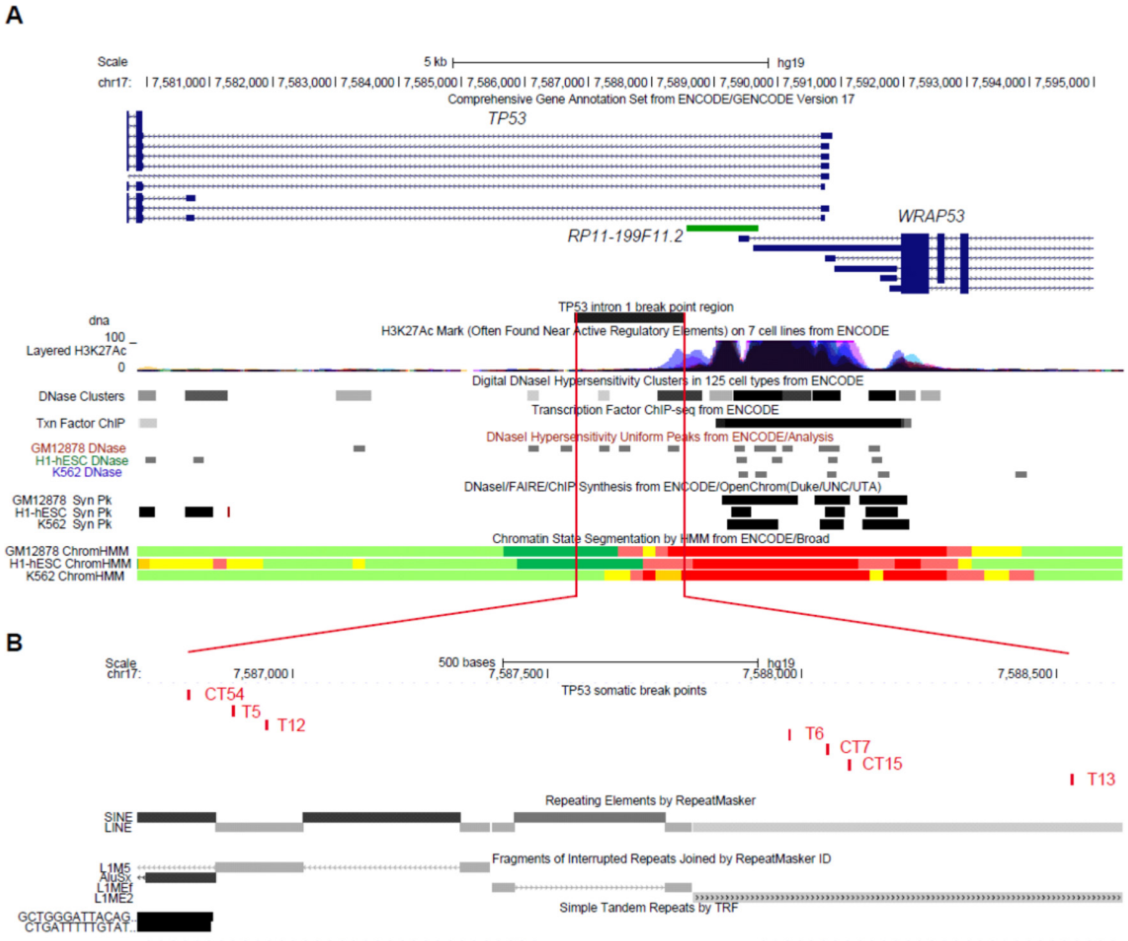


**Supplementary Figure S7: *TP53* break point cluster region is located near sites of active chromatin and DNA breaks occur in LINE sequences.** UCSC genome browser view (http://genome.ucsc.edu/; [2]) of the *TP53* intron 1 locus. **(A)** ENCODE epigenetic information is derived from the analysis of commonly used cell lines [3]. Tracks from top to bottom are: gene annotation after exclusion of 'problematic' transcripts; user track indicating the break point cluster region; histone 3 lysine 27 acetylation (H3K27ac) chromatin immunoprecipitation-sequencing (ChIP-seq) of seven cell lines as a superimposed layered track suggests active regulatory sites; DNaseI hypersensitive sites derived from ChIP-seq and a uniform peak analysis represent open chromatin; integrated active chromatin information prediction of DNaseI hypersensitivity, formaldehyde-assisted isolation of regulatory elements (FAIRE), and ChIP-seq of three cell lines; chromatin state segmentation analysis: dark red = active promoter, light red = weak promoter, orange = strong enhancer, yellow = weak/poised enhancer, dark green = transcriptional transition/elongation, light green = weak transcription. **(B)** *TP53* intron 1 break points are indicated by red vertical lines and IDs. Below are UCSC track information on repetitive DNA sequences.

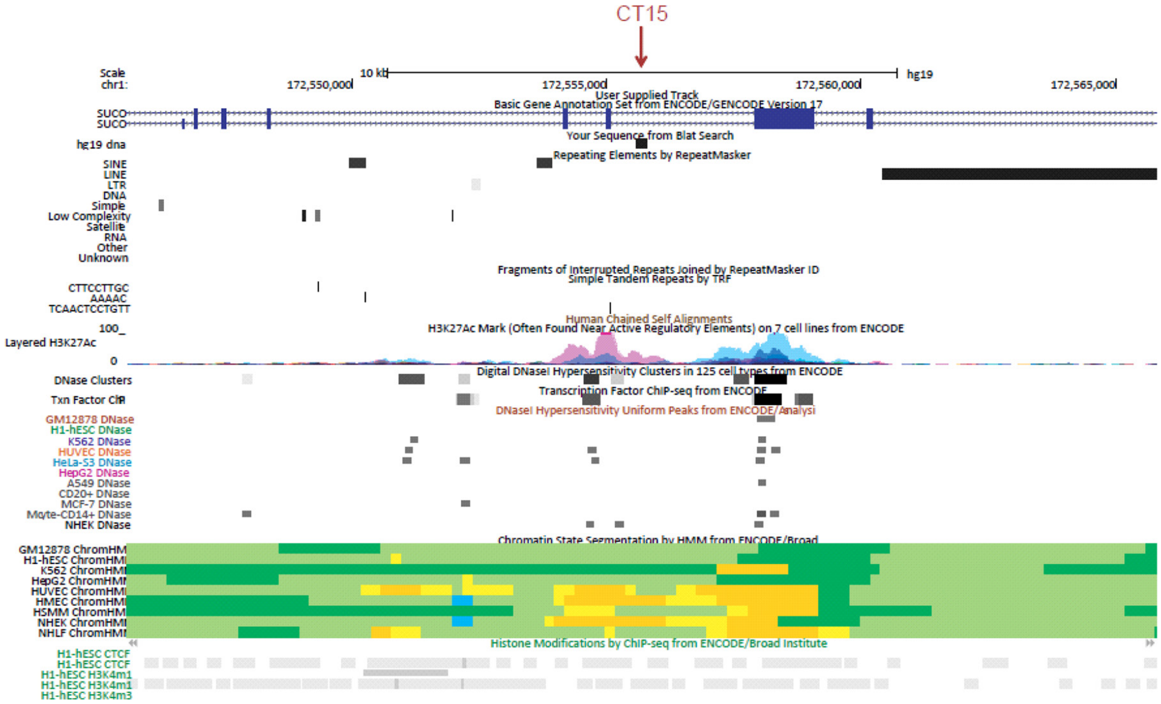


**Supplementary Figure S8: Regulatory information of translocation partner site on chromosome 1 of *TP53* translocation in OS patient KRD.** UCSC genome browser view of repetitive sequences and ENCODE regulatory information. Break point location is indicated by red arrow. For track description, see legend of Supplementary Figure S7.

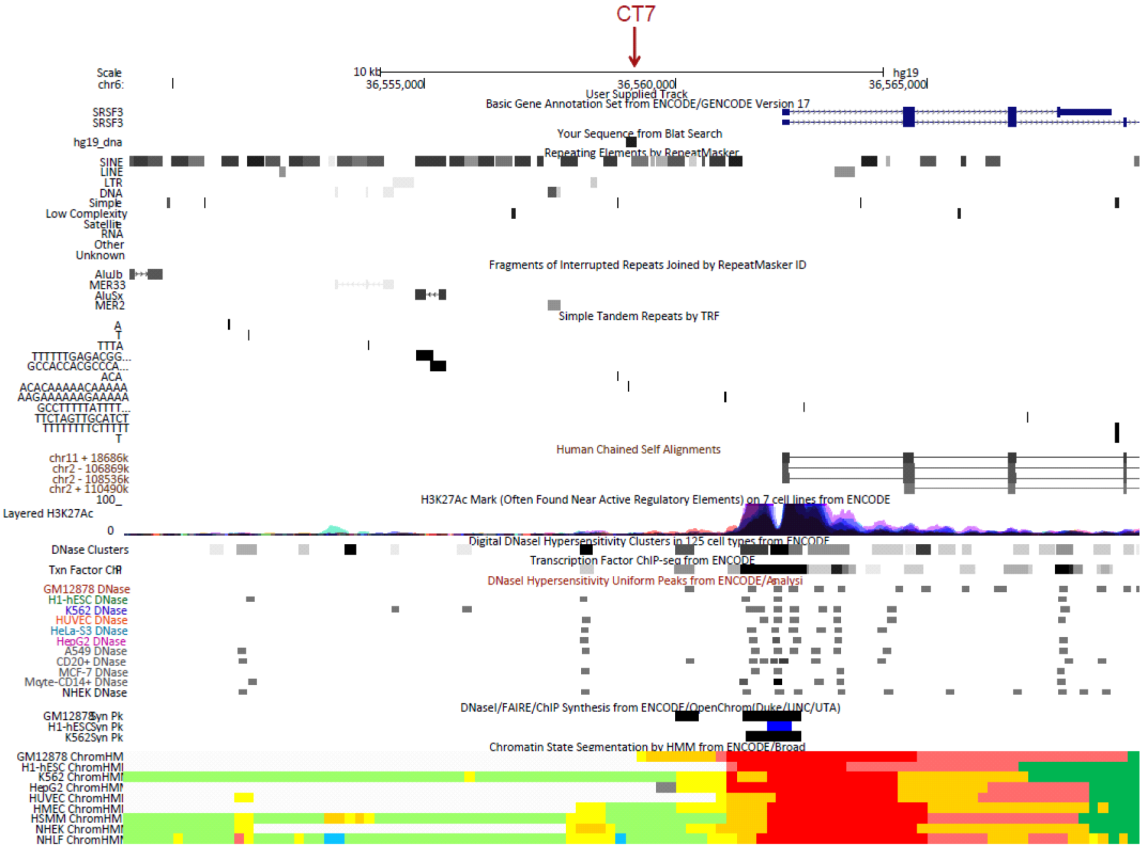


**Supplementary Figure S9: Regulatory information of translocation partner site on chromosome 6 of *TP53* translocation in OS patient KRD.** UCSC genome browser view of repetitive sequences and ENCODE regulatory information. Break point location is indicated by red arrow. For track description, see legend of Supplementary Figure S7.

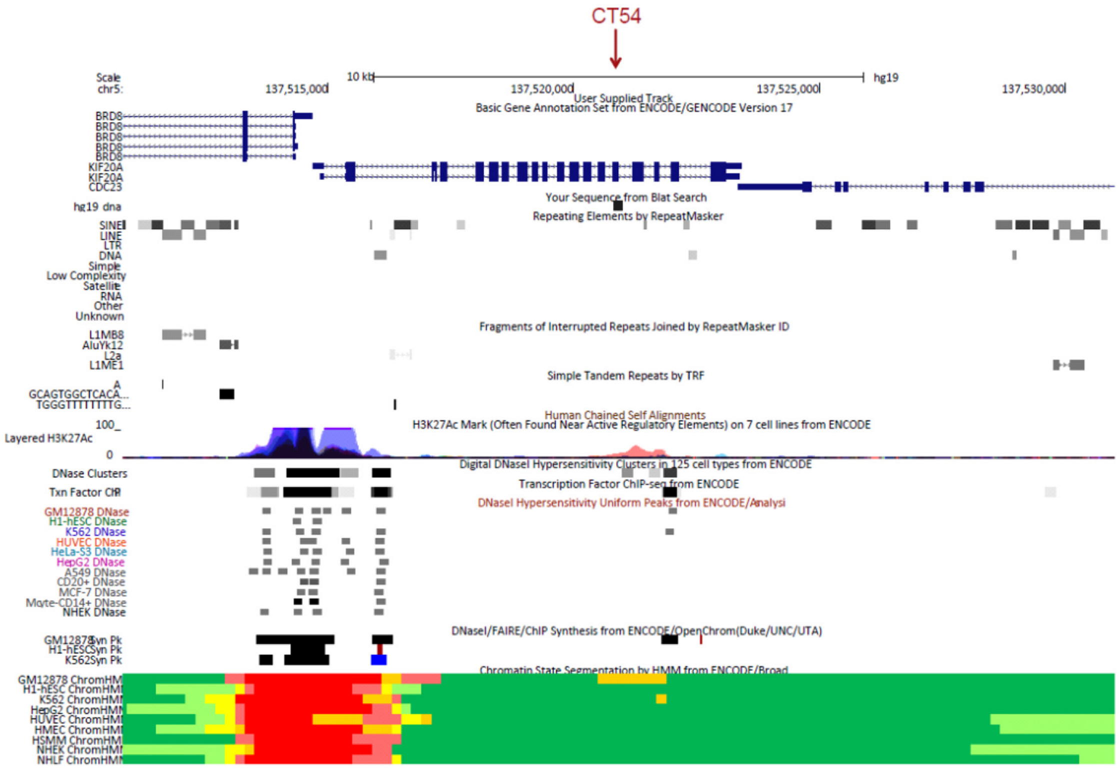


**Supplementary Figure S10: Regulatory information of translocation partner site on chromosome 5 of *TP53* translocation in OS patient KRD.** UCSC genome browser view of repetitive sequences and ENCODE regulatory information. Break point location is indicated by red arrow. For track description, see legend of Supplementary Figure S7.

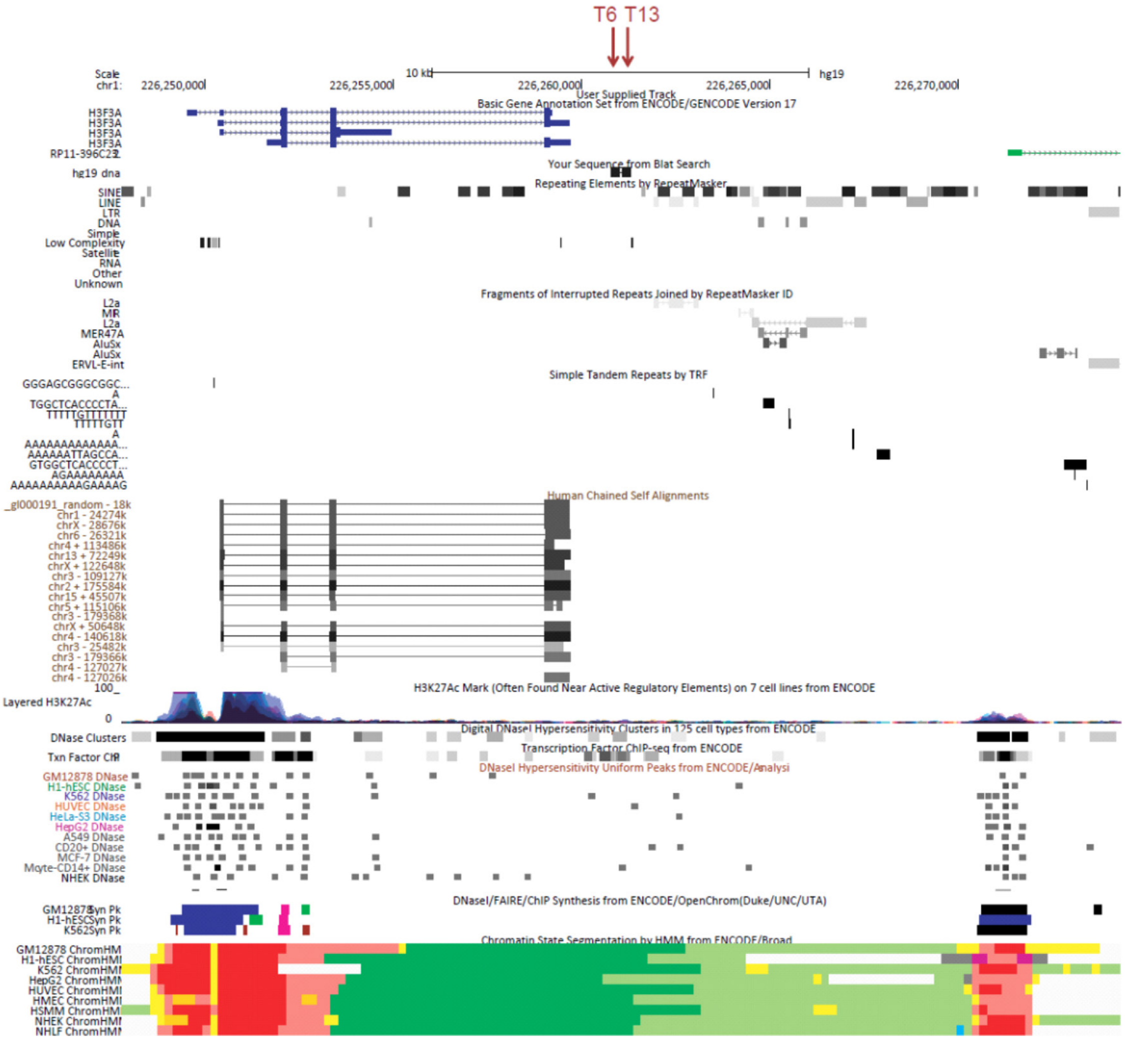


**Supplementary Figure S11: Regulatory information of partner site on chromosome 1 of *TP53* balanced translocation in OS patient YZH.** UCSC genome browser view of repetitive sequences and ENCODE regulatory information. Break point locations are indicated by red arrows. For track description, see legend of Supplementary Figure S7.

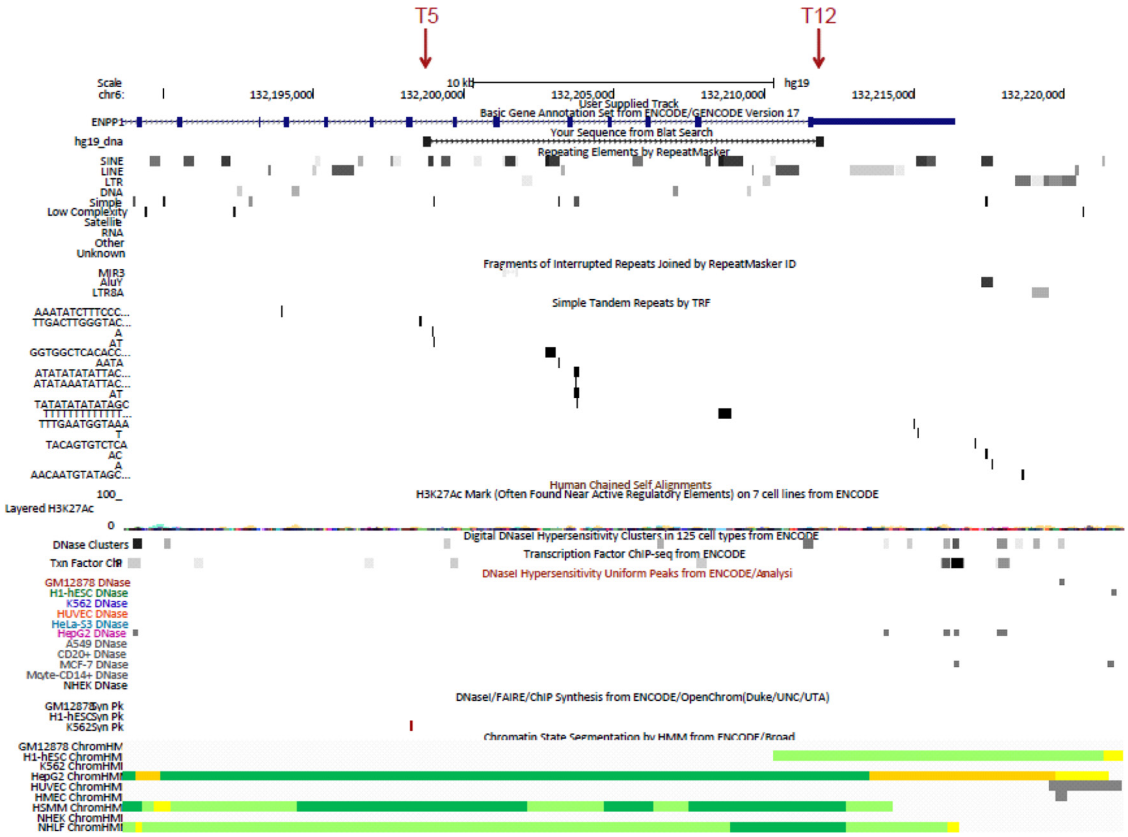


**Supplementary Figure S12: Regulatory information of genomic region on chromosome 6 which got inserted into *TP53* in OS patient PZP.** UCSC genome browser view of repetitive sequences and ENCODE regulatory information. Break points of the 12.5 kb segment which got inserted into intron 1 of *TP53* are indicated by red arrows. For track description, see legend of Supplementary Figure S7.

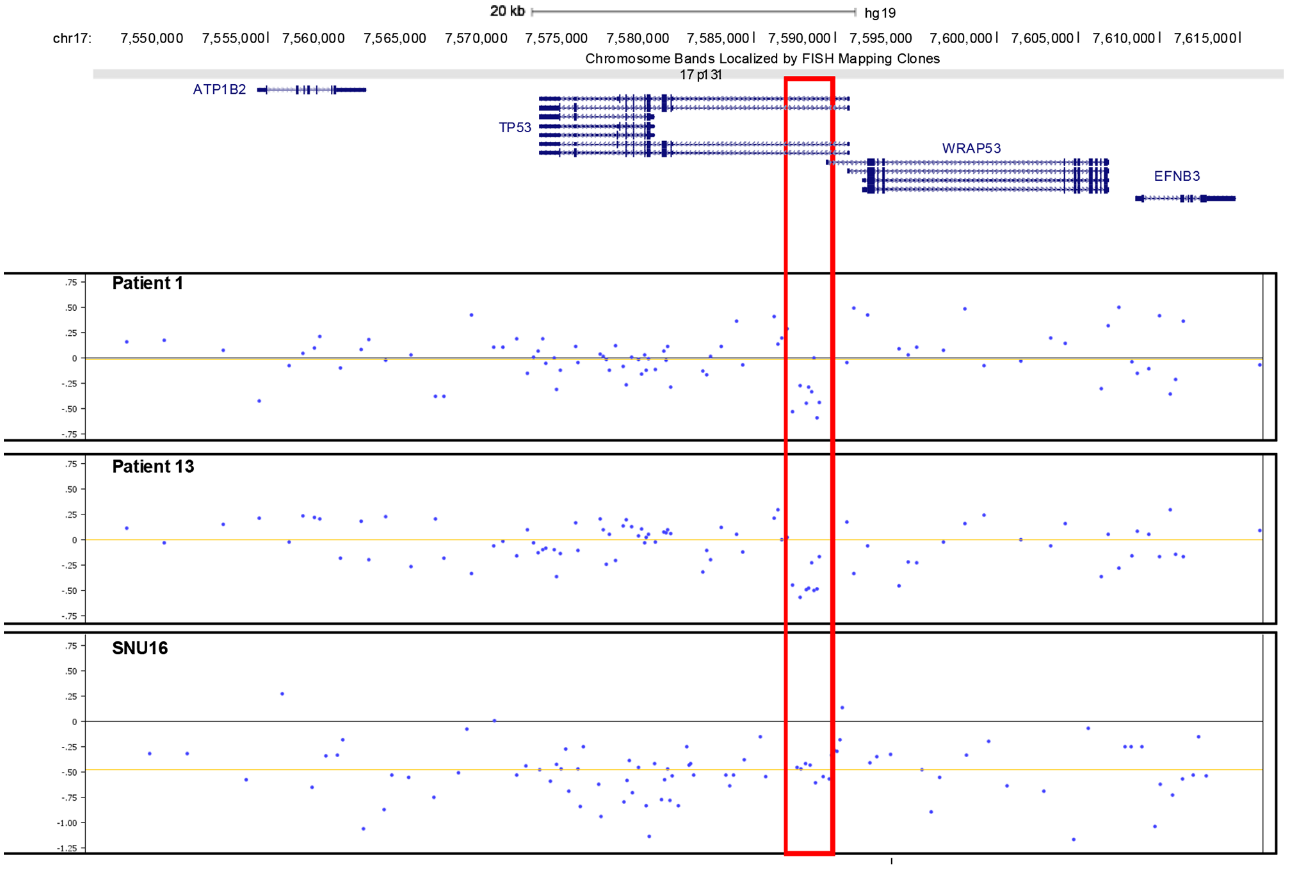


**Supplementary Figure S13: Copy number analysis of the *TP53* locus of LFS family members 1 and 13 by CytoScan array.** CytoScan array derived copy number analysis of LFS patients 1 and 13 and as copy number loss control gastric cancer cell line SNU16. Gene content of chr17:7,544,275..7,619,275 (NCBI genome build 37) is shown on top, log2 ratios of genomic marker intensities (y-axis) are displayed across the locus (x-axis). SNU16 has a heterozygous deletion of the entire locus (log2 ratio ca. 0.5) and intensities of seven markers of patients 1 and 13 show lower intensities (log2 ratio ca. 0.5, red box) which was not called by Chromosome Analysis Suite version CytoB-N1.2.2.271 (r4615; Affymetrix).

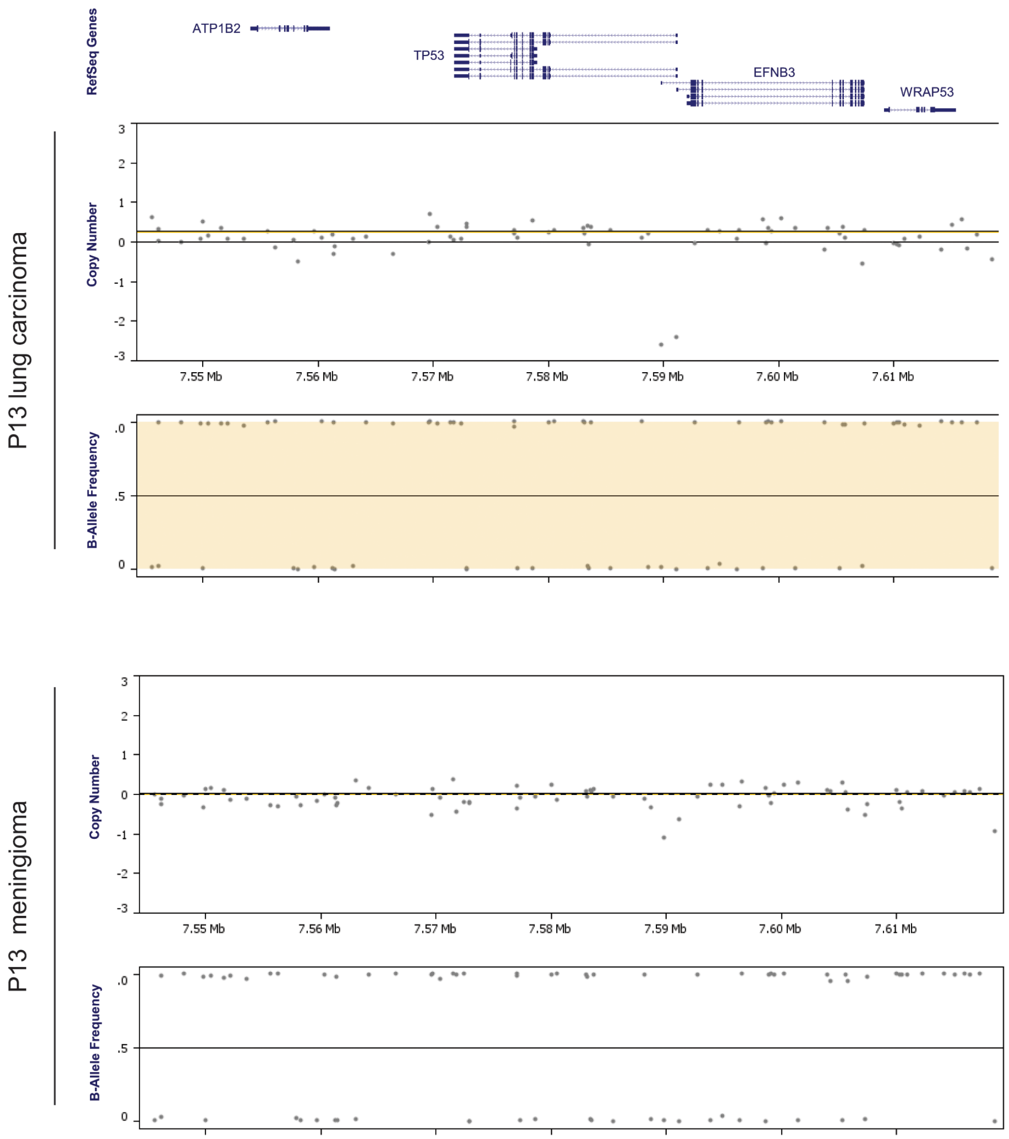


**Supplementary Figure S14: Copy number analysis of the *TP53* locus of two tumors of LFS family member P13 by OncoScan array.** OncoScan array derived copy number analysis of lung carcinoma and meningioma of LFS patient P13. Gene content of chr17:7,544,000..7,618,000 (NCBI genome build 37) is shown on top, log2 ratios of genomic marker intensities (y-axis) are displayed across the locus (x-axis). Copy number ratio relative to control indicates the presence of two chromosome copies and B allele frequency shows only homozygous variant calls with frequencies close to 1 or 0, respectively, indicating LOH.

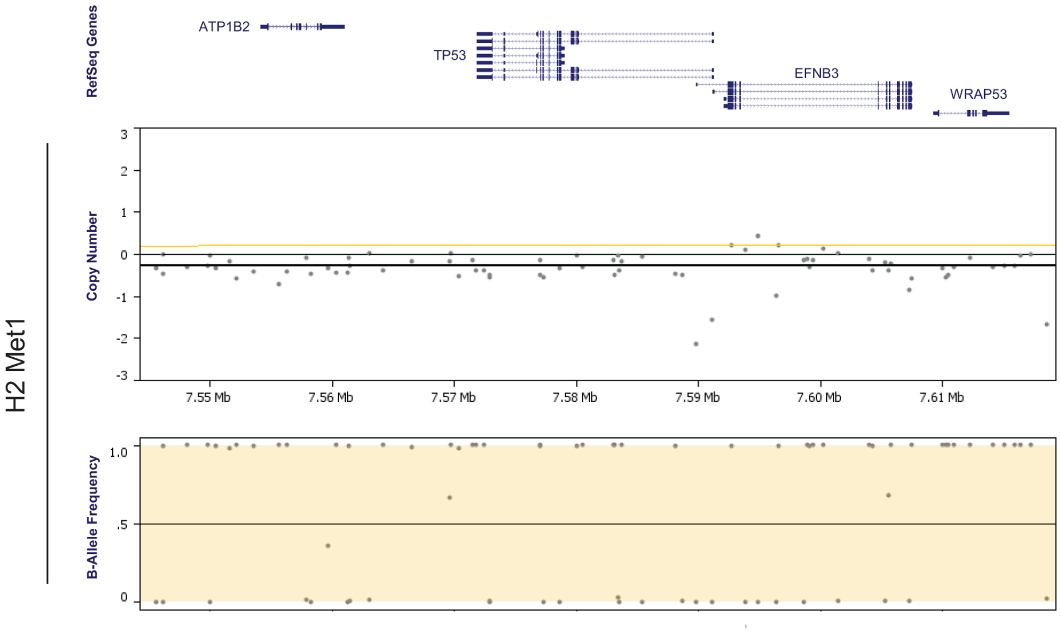


**Supplementary Figure S15: Copy number analysis of the *TP53* locus of metastasis of LFS family member H2 by OncoScan array.** OncoScan array display is as described for Supplementary Figure S14.

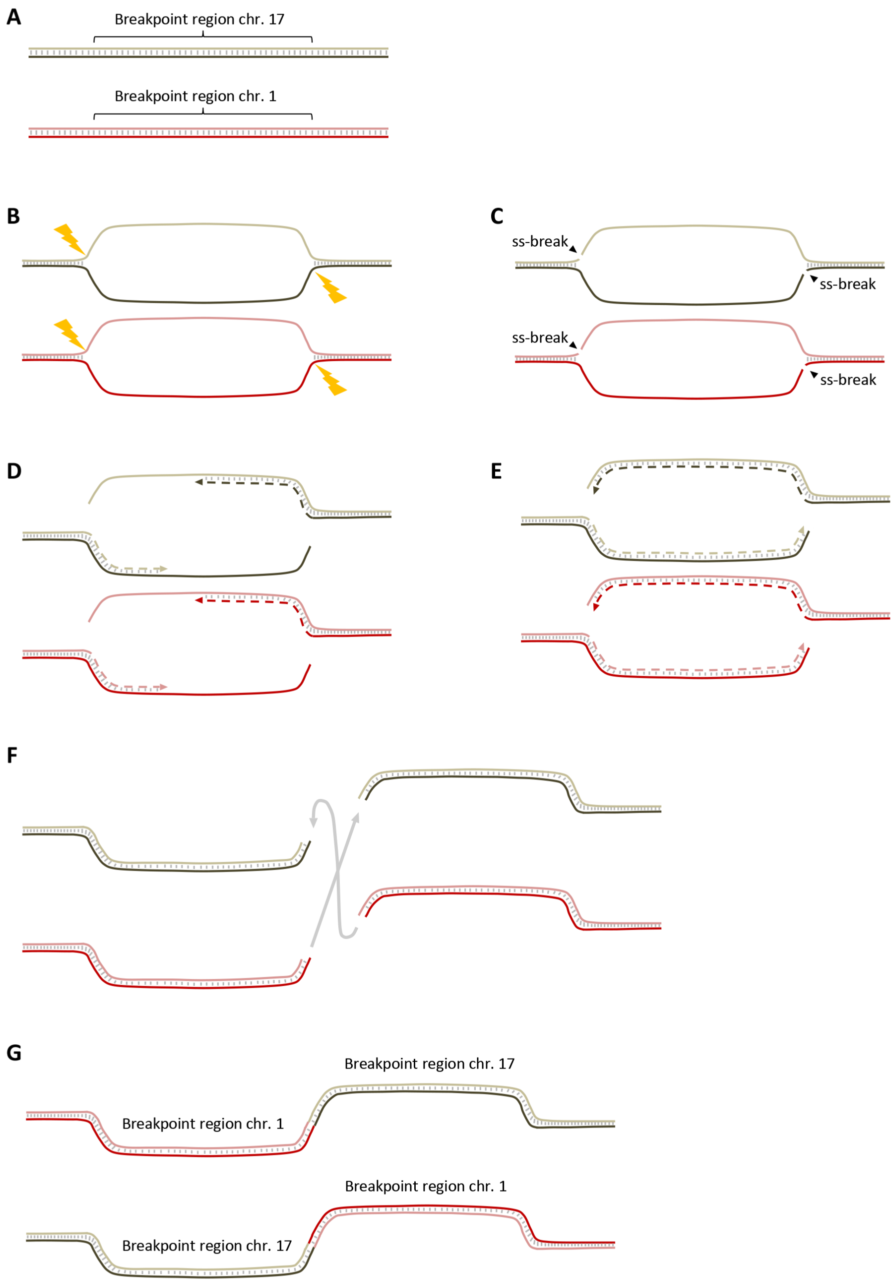


**Supplementary Figure S16: Hypothetical *TP53* intron 1 rearrangement mechanism in OS.** Schematic representation of a hypothetical rearrangement mechanism that can explain the duplication of 46 bp to 555 bp flanking the *TP53* intron 1 rearrangement points. We suggest that at a site of intense transcription, a large 'bubble' of two single DNA strands or an accumulation of more than one, maybe stalled, transcription bubbles occur. DNA breaks on both strands at different positions in the bubble(s) followed by end repair and NHEJ with other chromosomal partner sites. **(A)** to **(G)** chronological order of events. Single DNA strands are represented by light/dark green and red lines, respectively. DNA double strand pairing is represented by short gray lines. After opening of the DNA double strand, two single strand breaks occur that are not complementary to each other, i.e. that are at different sites of the bubble, resulting in a double strand break with about 50 to 550 bases of single strand 5' overhangs. Complementary strands are filled in 5' > 3' by the DNA repair machinery (dashed lines) resulting in a duplication of the single stranded segments. This process can take place at two different loci in the same cell (and same transcriptional hub) and can result in the fusion of different genomic regions by NHEJ **(F)**. **(G)** As a result, both reciprocal fusion products contain the same flanking sequences. ss-break, single strand DNA break. Of note, we observed only in patient YZH that also the non-*TP53* locus (chr. 1) shows duplication of break point-flanking sequences suggesting that the non-*TP53* translocation partner site can have no or short single strand overhangs.

## Supplementary Table S1: Sequencing statistics of four OS primary tumors by DNA-PET using the SOLiD platform

| Library | Sample | Total beads | Both tags mapped | Span [bp] | Within span | Within span (NR)[1] | Redun-dancy | Coverage[2] | dPETs[3] | dPET clusters (size 2+)[4] |
|---|---|---|---|---|---|---|---|---|---|---|
| DHO004HG19 | AJF | 172,897,363 | 92,577,911 | 1510-2570 | 89,073,088 | 66,594,042 | 1.34 | 46.85 | 1,383,636 | 2,278 |
| DHO005HG19 | PZP | 275,786,428 | 102,187,549 | 1910-3060 | 86,873,155 | 62,826,184 | 1.38 | 53.78 | 13,893,715 | 3,811 |
| DHO006DHO008HG19 | KRD | 286,596,700 | 168,109,213 | 660-3980 | 146,976,367 | 42,328,532 | 3.47 | 23.13 | 9,695,122 | 2,733 |
| DHO007DHO009HG19 | YZH | 378,703,399 | 167,518,527 | 1140-4030 | 147,613,747 | 60,005,658 | 2.46 | 44.98 | 10,336,682 | 3,707 |

1)non-redundant paired reads; exclusion of identical PCR amplicons
2)physical genome-wide coverage
3)discordant mapping paired-end tags
4)clusters of dPETs of which their tag one reads and tag 2 reads map to the same regions (regions A and B, respectively), connecting the same two regions

## Supplementary Table S2: Statistics of filtering of dPET cluster artifacts before identification of SVs

| | |
|---|---|
| dPET clusters of size ≥3 before artifact filtering | 4737 |
| Filter clusters with superclustersize >40 and UniqueClusterSize <6 | 218 |
| Blast1 score >2000 | 265 |
| Blast1 alignment type EC | 140 |
| MaxSelfChainLeftCov >80 | 240 |
| MaxSelfChainRightCov >80 | 6 |
| Match with simulated library | 226 |
| Exclude Overlap | 1 |
| dPET clusters of size ≥3 after artifact filtering | 3641 |

For explanation of individual steps see **Materials and Methods** and (Hillmer, Yao et al. 2011; Ng, Hillmer et al. 2012).

## Supplementary Table S3: Statistics of germline and somatic SVs identified in four OS tumors

| | DHO004HG19 | DHO005HG19 | DHO006DHO008HG19 | DHO007DHO009HG19 |
|---|---|---|---|---|
| | AJF | PZP | KRD | YZH |
| Deletion | 773 | 603 | 358 | 532 |
| Tandem duplication | 20 | 3 | 11 | 15 |
| Unpaired inversion | 87 | 72 | 92 | 110 |
| Isolated translocation | 49 | 53 | 129 | 80 |
| Inversion | 33 | 30 | 31 | 39 |
| Insertion | 18 | 30 | 17 | 27 |
| Complex rearrangement | 68 | 57 | 111 | 193 |
| Total | 1,048 | 848 | 749 | 996 |

## Supplementary Table S4: Statistics of somatic SVs identified in four OS tumors

| | DHO004HG19 | DHO005HG19 | DHO006DHO008HG19 | DHO007DHO009HG19 |
|---|---|---|---|---|
| | AJF | PZP | KRD | YZH |
| Deletion | 289 | 177 | 63 | 112 |
| Tandem duplication | 15 | 1 | 7 | 10 |
| Unpaired inversion | 56 | 46 | 64 | 89 |
| Isolated translocation | 34 | 36 | 119 | 72 |
| Inversion | 3 | 0 | 2 | 4 |
| Insertion | 9 | 6 | 2 | 4 |
| Complex rearrangement | 28 | 23 | 91 | 129 |
| Total | 434 | 289 | 348 | 420 |

## Supplementary Table S5: Statistics of genes affected by somatic SVs of four OS primary tumors

| Type of gene alteration | Number of genes |
|---|---|
| Genes in deletions (<1Mb) | 39 |
| Genes in tandem duplications (<1Mb) | 2 |
| Genes spanning SVs within one intron | 181 |
| Genes spanning SVs affecting coding sequences | 31 |
| Fusion genes | 67 |
| 5’ truncated genes | 265 |
| 3’ truncated genes | 329 |

## Supplementary Table S6: Somatic SVs of four OS primary tumors (Supplementary Data Set)

## Supplementary Table S7: Break point coordinates of *TP53* affecting rearrangements in four OS tumors

| Sample | SV ID | Strand left | Validated break left | Strand right | Validated break right | Structural variation | Truncated gene left (strand) | Truncated gene right (strand) | Predicted fusion gene or mode of truncation |
|---|---|---|---|---|---|---|---|---|---|
| AJF | D38 | + | chr17:7,557,506 | + | chr17:7,651,285 | deletion | *ATP1B2*(+) | *DNAH2*(+) | *ATP1B2-DNAH2* |
| KRD | CT54 | + | chr5:137,520,904 | + | chr17:7,586,797 | complex inter-chr. | *KIF20A*(+) | *TP53*(–) | 2 x 3’ truncation |
| KRD | CT7 | – | chr6:36,559,119 | + | chr17:7,588,049 | complex inter-chr. | – | *TP53*(–) | 3’ truncation |
| KRD | CT15 | + | chr1:172,555,688 | – | chr17:7,588,094 | complex inter-chr. | *SUCO*(+) | *TP53*(–) | *SUCO-TP53* |
| PZP | T5 | – | chr6:132,198,801[1)] | + | chr17:7,586,884[1)] | insertion inter-chr. | *ENPP1*(+) | *TP53*(–) | *TP53-ENPP1*[2)] |
| PZP | T12 | + | chr6:132,211,834 | – | chr17:7,586,948 | insertion inter-chr. | *ENPP1*(+) | *TP53*(–) | *ENPP1-TP53*[2)] |
| YZH | T6 | – | chr1:226,260,906 | + | chr17:7,587,974 | isolated translocation | – | *TP53*(–) | 3’ truncation |
| YZH | T13 | + | chr1:226,261,198 | – | chr17:7,588,529 | isolated translocation | – | *TP53*(–) | 5’ truncation |

[1)]DNA-PET mapping coordinates; rearrangement point could not be amplified by PCR
[2)]One fusion transcript based on an insertion of a part of *ENPP1* into *TP53* (see Figure S5)

## Supplementary Table S8: Bone-forming tumors or tumor-like lesions other than OS

| Diagnosis | *n* | Diagnosis | *n* |
|---|---|---|---|
| Fibrous dysplasia | 42/50 | Fibrocartilaginous mesenchymoma | 4/4 |
| Aneurysmal bone cyst | 14/17 | Nora lesion | 2/2 |
| Osteoid osteoma | 14/15 | Osteofibrous dysplasia | 2/2 |
| Ossifying fibroma | 13/15 | Osseous dysplasia | 2/2 |
| Reactive bone formation | 12/13 | Desmoplastic fibroma | 2/2 |
| Osteoblastoma | 8/8 | Adamantinoma | 1/1 |
| Myositis ossificans | 7/9 | Non-ossifying fibroma | 1/1 |

*n* = number of evaluable cases/total number of cases

## Supplementary Table S9: Overall survival of TMA OS patients stratified for *TP53* FISH signal and protein expression

| Overall survival | 5 years | 10 years |
|---|---|---|
| **TP53 FISH** | | |
| FISH negative (*n* = 192) | 61.20% | 58.70% |
| FISH positive (*n* = 23) | 64.90% | 64.90% |
| **TP53 immunhistochemistry** | | |
| IHC negative (*n* = 170) | 59.80% | 57.10% |
| IHC positive (*n* = 42) | 59.20% | 54.30% |

## Supplementary Table S10: TMA content and result of *TP53* FISH analysis

| Tissue type | *n* |
|---|---|
| Adrenal gland, adenoma | 4/4 |
| Brain, astrocytoma | 11/12 |
| Brain, glioblastoma | 15/17 |
| Brain, Meningeoma | 14/15 |
| Brain, normal | 1/1 |
| Brain, oligodendroglioma | 5/5 |
| Breast, ductal carcinoma | 18/20 |
| Breast, lobular carcinoma | 16/18 |
| Breast, medullary carcinoma | 21/24 |
| Breast, mucinous carcinoma | 7/8 |
| Breast, normal | 1/1 |
| Breast, tubular carcinoma | 8/9 |
| Cervix, *in situ* carcinoma | 10/11 |
| Colon, adenoma | 41/46 |
| Colon, carcinoma | 21/24 |
| Colon, normal | 0/0 |
| Endometrium, endometrioid carcinoma | 12/13 |
| Endometrium, normal | 1/1 |
| Endometrium, serous carcinoma | 11/11 |
| Esophagus, adenocarcinoma | 3/3 |
| Esophagus, normal | 2/2 |
| Esophagus, small cell carcinoma | 1/1 |
| Esophagus, squamous cell carcinoma | 7/8 |
| Fat tissue, normal | 0/0 |
| Gall bladder, carcinoma | 12/13 |
| Gall bladder, normal | 5/6 |
| Heart, normal | 4/4 |

(*Continued*)

| Tissue type | *n* |
|---|---|
| Kidney, chromophobe carcinoma | 2/3 |
| Kidney, clear cell carcinoma | 24/27 |
| Kidney, normal | 3/3 |
| Kidney, oncocytoma | 8/8 |
| Kidney, papillary carcinoma | 11/12 |
| Larynx, carcinoma | 13/14 |
| Liver, hepatocellular carcinoma | 28(32 |
| Liver, normal | 5/5 |
| Lung, adenocarcinoma | 54/61 |
| Lung, large cell carcinoma | 15/17 |
| Lung, normal | 4/5 |
| Lung, small cell carcinoma | 15/15 |
| Lung, squamous cell carcinoma | 30/32 |
| Lymph node, Hodgkin Lymphoma | 15/18 |
| Lymph node, non Hodgkin Lymphoma | 18/19 |
| Lymph node, normal | 2/2 |
| Mesothelioma | 13/14 |
| Myometrium, normal | 2/2 |
| Myometrium, myoma | 18/22 |
| Nerve, neurofibroma | 12/14 |
| Nerve, schwannoma | 8/9 |
| Oral cavity, carcinoma | 18/21 |
| Oral cavity, normal | 5/5 |
| Ovary, endometrioid carcinoma | 18/20 |
| Ovary, mucinous carcinoma | 3/3 |
| Ovary, normal | 0/0 |
| Ovary, serous carcinoma | 17/18 |
| Pancreas, adenocarcinoma | 14/15 |
| Pancreas, normal tissue | 4/6 |
| Paraganglioma | 5/6 |
| Parathyroid, adenoma | 17/21 |
| Parathyroid, normal | 0/0 |
| Pheochromozytoma | 10/11 |
| Prostate, adenocarcinoma | 33/37 |
| Prostate, normal | 9/9 |
| Salivary gland, cylindroma | 11/11 |
| Salivary gland, normal | 6/6 |

(*Continued*)

| Tissue type | *n* |
|---|---|
| Salivary gland, pleomorphic adenoma | 16/18 |
| Salivary gland, Warthin tumor | 7/8 |
| Skeletal muscle, normal | 2/3 |
| Skin, basal cell carcinoma | 23/27 |
| Skin, histiocytoma | 9/10 |
| Skin, Kaposi sarcoma | 8/9 |
| Skin, melanoma | 32/36 |
| Skin, naevus | 22/24 |
| Skin, normal | 4/4 |
| Skin, squamous cell carcinoma | 10/10 |
| Small intestine carcinoma | 8/9 |
| Small intestine, normal | 0/0 |
| Soft tissue, giant cell tumor of tendon sheath | 10/11 |
| Soft tissue, hemangioma, capillary type | 6/6 |
| Soft tissue, leiomyosarcoma | 13/15 |
| Soft tissue, lipoma | 5/5 |
| Soft tissue, liposarcoma | 6/6 |
| Soft tissue, pleomorphic sarcoma | 9/10 |
| Stomach, carcinoma, diffuse type | 7/8 |
| Stomach, carcinoma, intestinal type | 14/17 |
| Stomach, normal | 4/5 |
| Testis, non-seminomatous carcinoma | 16/20 |
| Testis, normal | 3/3 |
| Testis, seminoma | 14/14 |
| Thymus, normal | 1/1 |
| Thymus, thymoma | 8/8 |
| Thyroid, adenoma | 11/12 |
| Thyroid, follicular carcinoma | 12/13 |
| Thyroid, normal | 1/1 |
| Thyroid, papillary carcinoma | 5/5 |
| Urinary bladder, invasive carcinoma | 10/11 |
| Urinary bladder, non-invasive carcinoma | 10/10 |
| Urinary bladder, normal | 15/16 |
| Vulva, squamous cell carcinoma | 8/8 |
| **Total tumor** | **966/1072** |
| **Total normal** | **84/91** |
| **Total tumor + normal** | **1,050/1,163** |

*n* = number of evaluable cases/total number of cases

## Supplementary Table S11: Summary of TMA *TP53* FISH analysis

| Kind of tissue | *n* |
|---|---|
| Normal tissue | 84/91 |
| Brain tumors | 45/49 |
| Breast tumors | 70/79 |
| Gynecologic tumors | 97/106 |
| Genitourinary tumors | 128/142 |
| Others | 118/131 |
| Gastrointestinal tumors | 208/234 |
| Lung tumors | 114/125 |
| Lymphoid tumors | 33/37 |
| Skin tumors | 104/116 |
| Soft tissue tumors | 49/53 |
| **Total** | **1,050/1,163** |

*n* = number of evaluable cases/total number of cases

## Supplementary Table S12: Copy number variations of two affected LFS family members based on CytoScan array analysis

| Patient | CN[1] State | Type | Chr | Min[2] | Max[2] | Size (kb) | Marker Count | Confidence | Genes | Comment for CNV shared between patient 1 and 2 |
|---|---|---|---|---|---|---|---|---|---|---|
| 1 | 0 | Loss | 2 | 41,239,606 | 41,250,106 | 10.5 | 5 | 0.9954 | | |
| 1 | 1 | Loss | 2 | 168,172,736 | 168,180,626 | 7.89 | 11 | 0.9297 | | |
| 13 | 1 | Loss | 2 | 168,172,736 | 168,180,626 | 7.89 | 11 | 0.9623 | | |
| 1 | 1 | Loss | 3 | 160,772,598 | 160,778,822 | 6.224 | 9 | 0.9388 | PPM1L | |
| 13 | 3 | Gain | 4 | 132,780,784 | 132,894,269 | 113.485 | 45 | 0.8855 | | |
| 1 | 3 | Gain | 5 | 32,108,414 | 32,162,756 | 54.342 | 53 | 0.9247 | PDZD2, GOLPH3 | CNV site in DGV |
| 13 | 3 | Gain | 5 | 32,108,414 | 32,166,970 | 58.556 | 55 | 0.9186 | PDZD2, GOLPH3 | CNV site in DGV |
| 13 | 1 | Loss | 7 | 3,833,336 | 3,852,657 | 19.321 | 14 | 0.9512 | SDK1 | |
| 13 | 0 | Loss | 8 | 39,234,303 | 39,357,501 | 123.198 | 8 | 1.0000 | ADAM5P, ADAM3A | |
| 13 | 1 | Loss | 8 | 84,367,320 | 84,380,551 | 13.231 | 11 | 0.9519 | | |
| 1 | 1 | Loss | 8 | 96,076,737 | 96,098,249 | 21.512 | 14 | 0.9106 | MIR3150 | CNV site in DGV |
| 13 | 1 | Loss | 8 | 96,076,737 | 96,098,249 | 21.512 | 14 | 0.9538 | MIR3150 | CNV site in DGV |
| 13 | 3 | Gain | 12 | 32,005,944 | 32,062,001 | 56.057 | 40 | 0.9289 | | |
| 1 | 1 | Loss | 12 | 69,023,587 | 69,032,212 | 8.625 | 9 | 0.9250 | RAP1B | |
| 13 | 3 | Gain | 14 | 106,022,513 | 107,051,759 | 1029.25 | 23 | 0.9158 | KIAA0125, ADAM6, NCRNA00226, NCRNA00221 | |

(*Continued*)

| Patient | CN[1] State | Type | Chr | Min[2] | Max[2] | Size (kb) | Marker Count | Confidence | Genes | Comment for CNV shared between patient 1 and 2 |
|---|---|---|---|---|---|---|---|---|---|---|
| 13 | 1 | Loss | 15 | 20,175,623 | 22,504,198 | 2328.58 | 48 | 0.8801 | HERC2P3, GOLGA6L6, GOLGA8C, BCL8, POTEB, NF1P1, LOC646214, CXADRP2, LOC727924, OR4M2, OR4N4, OR4N3P | CNV site in DGV |
| 1 | 1 | Loss | 15 | 22,300,190 | 22,504,198 | 204.008 | 38 | 0.8682 | LOC727924, OR4M2, OR4N4, OR4N3P | CNV site in DGV |
| 13 | 3 | Gain | 16 | 34,455,753 | 34,762,298 | 306.545 | 84 | 0.9135 | LOC283914, LOC146481, LOC100130700 | |
| 13 | 1 | Loss | 17 | 28,303,998 | 28,317,131 | 13.133 | 18 | 0.9198 | EFCAB5 | |
| 1 | 1 | Loss | 18 | 1,722,426 | 1,838,901 | 116.475 | 110 | 0.9158 | | |
| 13 | 3 | Gain | 18 | 51,345,688 | 51,423,648 | 77.96 | 57 | 0.9415 | | |
| 13 | 3 | Gain | 19 | 20,833,215 | 20,987,762 | 154.547 | 123 | 0.9063 | ZNF626 | |
| 13 | 1 | Loss | 19 | 28,294,543 | 28,318,358 | 23.815 | 15 | 0.9048 | | |
| 13 | 3 | Gain | 19 | 58,364,597 | 58,383,708 | 19.111 | 20 | 0.9187 | ZNF587, ZNF814 | |
| 1 | 1 | Loss | 20 | 32,806,979 | 32,822,219 | 15.24 | 9 | 0.9050 | | |
| 13 | 3 | Gain | 22 | 22,861,993 | 23,358,013 | 496.02 | 65 | 0.9360 | ZNF280B, ZNF280A, PRAME, LOC648691, POM121L1P, GGTLC2, MIR650, IGLL5 | |
| 1 | 2 | Gain | X | 2,706,943 | 2,713,139 | 6.196 | 2 | 0.2191 | XG | |
| 1 | 2 | Gain | X | 58,094,436 | 62,058,620 | 3964.18 | 19 | 0.2466 | | |
| 1 | 4 | Gain | X | 150,192,436 | 150,195,253 | 2.817 | 8 | 0.9315 | | |

[1]Copy number
[2]Position based on NCBI human reference Build 37

## REFERENCES


1. Krzywinski M, Schein J, Birol I, Connors J, Gascoyne R, Horsman D, Jones SJ, Marra MA. Circos: an information aesthetic for comparative genomics. Genome Research. 2009; 19:1639–1645.
2. Rhead B, Karolchik D, Kuhn RM, Hinrichs AS, Zweig AS, Fujita P a, Diekhans M, Smith KE, Rosenbloom KR, Raney BJ, Pohl A, Pheasant M, Meyer LR, et al. The UCSC Genome Browser database: update 2010. Nucleic Acids Research. 2010; 38:D613–619.
3. Djebali S, Davis C, Merkel A, Dobin A. Landscape of transcription in human cells. Nature. 2012; 489:101–108.
